# Urea-functionalized amorphous calcium phosphate nanofertilizers: optimizing the synthetic strategy towards environmental sustainability and manufacturing costs

**DOI:** 10.1038/s41598-021-83048-9

**Published:** 2021-02-09

**Authors:** Francisco J. Carmona, Gregorio Dal Sasso, Gloria B. Ramírez-Rodríguez, Youry Pii, José Manuel Delgado-López, Antonietta Guagliardi, Norberto Masciocchi

**Affiliations:** 1 Department of Science and High Technology and To.Sca.Lab, University of Insubria, Via Valleggio 11, 22100 Como, Italy; 2 Institute of Crystallography and To.Sca.Lab, Consiglio Nazionale Delle Ricerche, Via Valleggio 11, 22100 Como, Italy; 3 Department of Inorganic Chemistry, University of Granada, Av. Fuentenueva S/N, 18071 Granada, Spain; 4 Faculty of Science and Technologies, Free University of Bolzano, Piazza Università 5, 39100 Bolzano, Italy

**Keywords:** Nanoparticle synthesis, Nanoparticles

## Abstract

Nanosized fertilizers are the new frontier of nanotechnology towards a sustainable agriculture. Here, an efficient N-nanofertilizer is obtained by post-synthetic modification (PSM) of nitrate-doped amorphous calcium phosphate (ACP) nanoparticles (NPs) with urea. The unwasteful PSM protocol leads to N-payloads as large as 8.1 w/w%, is well replicated by using inexpensive technical-grade reagents for cost-effective up-scaling and moderately favours urea release slowdown. Using the PSM approach, the N amount is *ca.* 3 times larger than that obtained in an equivalent one-pot synthesis where urea and nitrate are jointly added during the NPs preparation. In vivo tests on cucumber plants in hydroponic conditions show that N-doped ACP NPs, with half absolute N-content than in conventional urea treatment, promote the formation of an equivalent amount of root and shoot biomass, without nitrogen depletion. The high nitrogen use efficiency (up to 69%) and a cost-effective preparation method support the sustainable real usage of N-doped ACP as a nanofertilizer.

## Introduction

By the adoption of the 2030 Agenda for Sustainable Development, the United Nations aim at ensuring food security through a more sustainable agriculture. The scenario opens the way to the need of increasing crop yields (to face the steadily growth of the global population jointly to the paucity of arable lands) and to a parallel and optimized management of the natural resources. For example, nitrogen (N), one of the most important macronutrients to plants together with phosphorus (P) and potassium (K), is a heavily spread element in the agricultural field. However, current practices are highly inefficient (more than 50% of N applied is lost by leaching, run-off and volatilization)^1,2^ and result in economic losses and a deleterious impact on the environment (eutrophication of inland water basins, atmosphere pollution with greenhouse gases, wastes of non-renewable P resources)^3,4^. New approaches enhancing the efficiency of plant fertilization and mitigating the adverse environmental consequences include the use of biodegradable polymer coatings from renewable resources^5^, N-release / plant uptake synchronization^6^ or rhizosphere microbiota assistance^7^, all methods relying on a relatively accurate control of the macronutrient supply rate to plants.

Recently, great attention is being given to the potential use of nanotechnology for improving plant nutrition and/or limiting undesired environmental effects^8–10^. Nanomaterials which are themselves composed of macronutrients can lead to soft(er) release of important molecules or ions, regulated by solubility and solubility rates that are very different from both those of the corresponding bulk and those of conventional fertilizers. Moreover, their higher surface area makes it possible to attain a higher loading of additional macronutrients (*e.g.* N in the form of nitrate or urea), offering different kinetic release profiles than their conventional counterparts^11^. Scientific papers and industrial patents have indeed proposed the usage of nanosized calcium (ortho)phosphate (CaP), of significantly reduced solubility, as new material providing encouraging results in agriculture, being it intrinsically rich in phosphorus and, after N-functionalization, also capable of delivering nitrogen as a second important macronutrient^12–19^. The advantage of saving non-renewable resources (phosphate rocks) for P-fertilization is intrinsic with CaP NPs, whereas maximizing the efficiency of N-doping and N-release is the major goal of NPs chemical preparation strategies. Nonetheless, the cost of NPs manufacturing remains typically overlooked, despite of the narrow profit margins available in the agricultural field which profoundly contrast those of high-tech and nanotechnology industries. Here we present an optimized strategy increasing the N-payloads of Amorphous Calcium Phosphate (ACP) NPs and consider the cost-effectiveness of an unwasteful preparation process, favoring reduction of environmental risks associated to unwanted material losses.

CaP materials encompass a broad list of crystalline phases characterized by Ca/P ratio ranging from 0.5 to 2.0, different hydration and phosphate protonation levels, water solubility, pH stability ranges, and polymorphic phases. A significant trend in thermodynamic solubility values with Ca/P ratio was found^20^, which limits the candidates usable in nearly neutral or slightly acidic conditions (such as those found in plant leaves and stems). Among these we mention: monetite (anhydrous dicalcium phosphate), OCP (octacalciumphosphate), TCP (tricalciumphosphate, α and β polymorphs) and (hydroxy)apatite (HAp)^21^. As monetite is stable only above 100 °C, OCP is a transient (thermodynamically unstable) species and neither form of TCP can be precipitated from aqueous solutions, HAp remains the most attractive candidate. In this respect, nanocrystalline (hydroxy)apatite (HAp) (the mineral component of bone and teeth in vertebrates)^22–24^ has long been studied in the biomedical and catalysis fields for a number of already marketed products^25,26^. Taking inspiration from these applications, nearly all investigations reported so far for synthetic CaP to be used as nanofertilizers focus on crystalline materials, mimicking the biogenic and non-toxic apatitic mineral component in bone.

Though of high relevance, the ability of ACP NPs to load nitrogen compounds, namely nitrate ions and urea molecules, and to act as a multifunctional nanofertilizer, remains poorly investigated. Attracted by the topic, we have recently studied ACP as a platform alternative to HAp NPs^27^. Nanosized ACP, of similar Ca/P content to HAp, possesses a higher surface reactivity, a more pronounced solubility (favored by the lower Ca:P ratio and presence of native or deliberately added carbonate ions)^20^, still much lower than that of conventional fertilizers, and a superior tendency to adsorb small molecules (as urea) on the NP surface than nanocrystalline apatite, thus offering higher N-payloads^27^. Noteworthy, carbonate is known to hamper the transformation of ACP into crystalline apatite in aqueous solution, justifying its use during ACP preparation^28^. On the other hand, ACP can be obtained at very short precipitation times and at lower temperature in comparison to (nano)crystalline CaP^26^. This is highly relevant for the cost-effective production of the nanomaterials for industrial exploitation. All these pieces of evidence highlight the superior features showed by ACP than HAp toward its use as a multinutrient nanofertilizer. Indeed, we recently developed a one-pot synthesis of N-doped ACP NPs by using excess nitrate and urea^27^. When supplied to *Triticum durum* plants, growth chamber experiments demonstrated the ability of the nanofertilizer to preserve yields and quality of the wheat grains in comparison to conventional fertilization protocols^27,29^. Likewise, foliar application to grapevines resulted in a high nitrogen use efficiency (NUE): with a nitrogen amount 15 times lower than in the conventional urea treatment, grapes with similar content in amino acids, beneficial to wine aroma and taste, were obtained^30^. Despite the promising results, the amount of nitrogen incorporated within the N-doped ACP-NPs by the one-pot synthesis did not exceed 2.8 w/w%, making this nanomaterial cost-ineffective for extensive applications.

Hereafter we present an alternative chemical approach towards a highly efficient post-synthetic modification (PSM) of ACP NPs with urea, leading to an N-doping level nearly 3 × higher than in the one-pot synthesis and with no waste of the added urea. We also demonstrate that the protocol efficiency is maintained by using tap water and inexpensive technical grade (TG) materials (bought from commercial suppliers of large-scale commodities), thus favoring the development of cost-effective and environmentally sustainable nanofertilizers preparation at a larger scale.

The potential applications of the N-doped ACP nanofertilizer include soil and foliar fertilization and soilless (hydroponic) cultivation conditions. Here, we selected hydroponic conditions since drainage water recycling and reduction of nutrient losses to the environment are therein ensured; we present a case of study by investigating the NPs functional behaviour when supplied to plants of cucumber (*Cucumis sativus*). Compared to recently reported applications of N-doped nanofertilizers in conventional field tests, soilless conditions may open a bright future for urban and vertical farming^31^, and new roads to increase productivity^32^, particularly in dry and heavily populated areas where arable lands are scarce^33^.

## Results and discussion

### A facile route towards maximizing the N-payload of ACP NPs

**Scheme 1:**
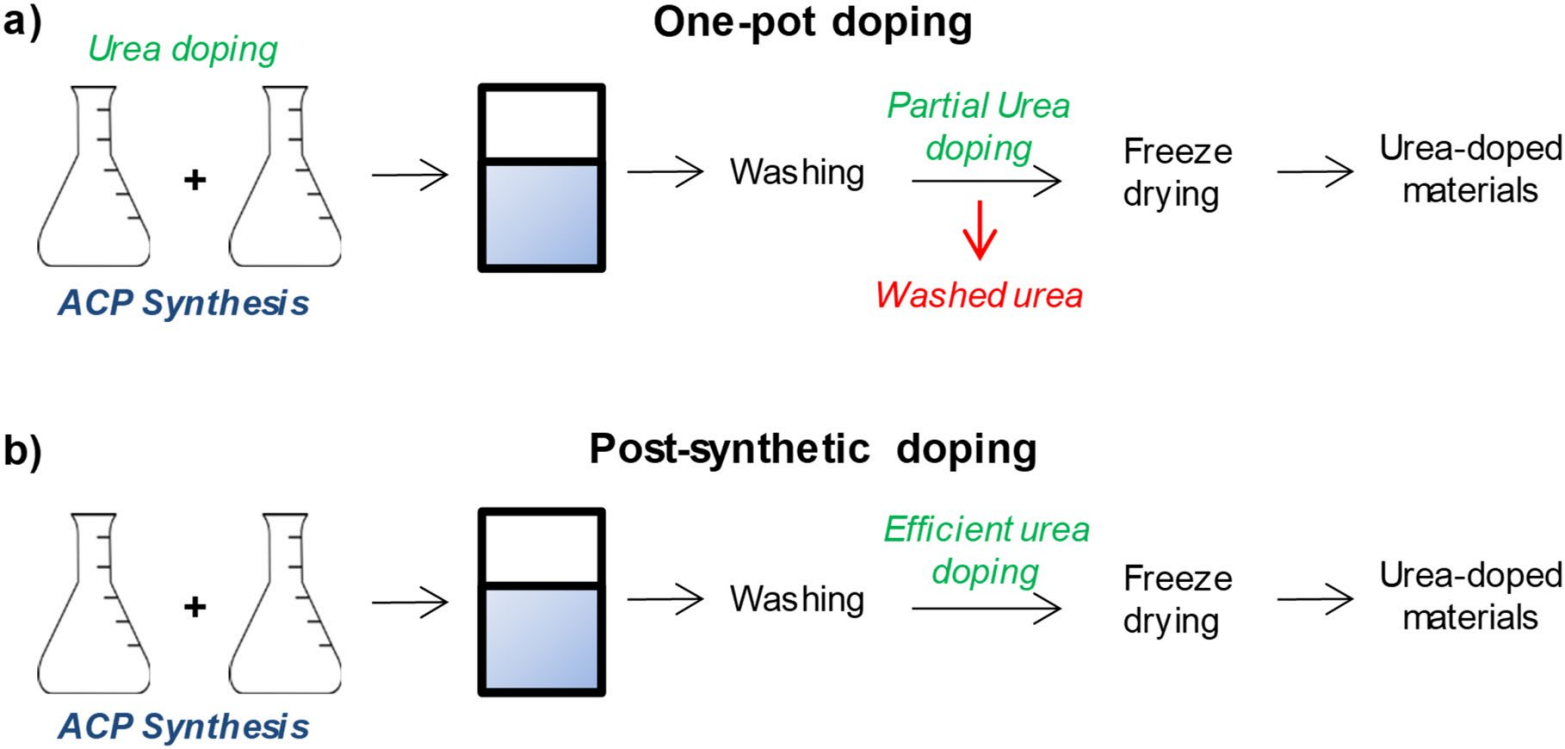
Schematic representation of the two different approaches for doping ACP NPs with urea: a) one-pot doping during the material preparation, losing weakly adsorbed urea during the NPs washing, and b) post-synthetic doping after the ACP NPs isolation and washing.

A screening with analytical grade reagents was firstly performed to identify the maximum amount of urea which could be adsorbed by the NPs, avoiding co-precipitation of urea or of other segregated crystalline phases. In these preparations, urea was added after the ACP NPs were precipitated, instead of during their one-pot synthesis^27^ (see Scheme 1). Conventional routes to coat NPs with valuable functional compounds in different fields (*e.g.* agriculture, nanomedicine, catalysis) typically imply a washing step to remove non-adsorbed, or weakly adsorbed, reactants. By the PSM approach proposed here, the washing step after urea functionalization is avoided. This alternative procedure ensures that the whole amount of urea added remains bound to the NPs surface and/or occluded in the functionalized materials, avoiding unwanted losses and, therefore, increasing enormously the efficiency of the doping process. Typical N loadings of 6.5% or above were reached.

**Scheme 2:**
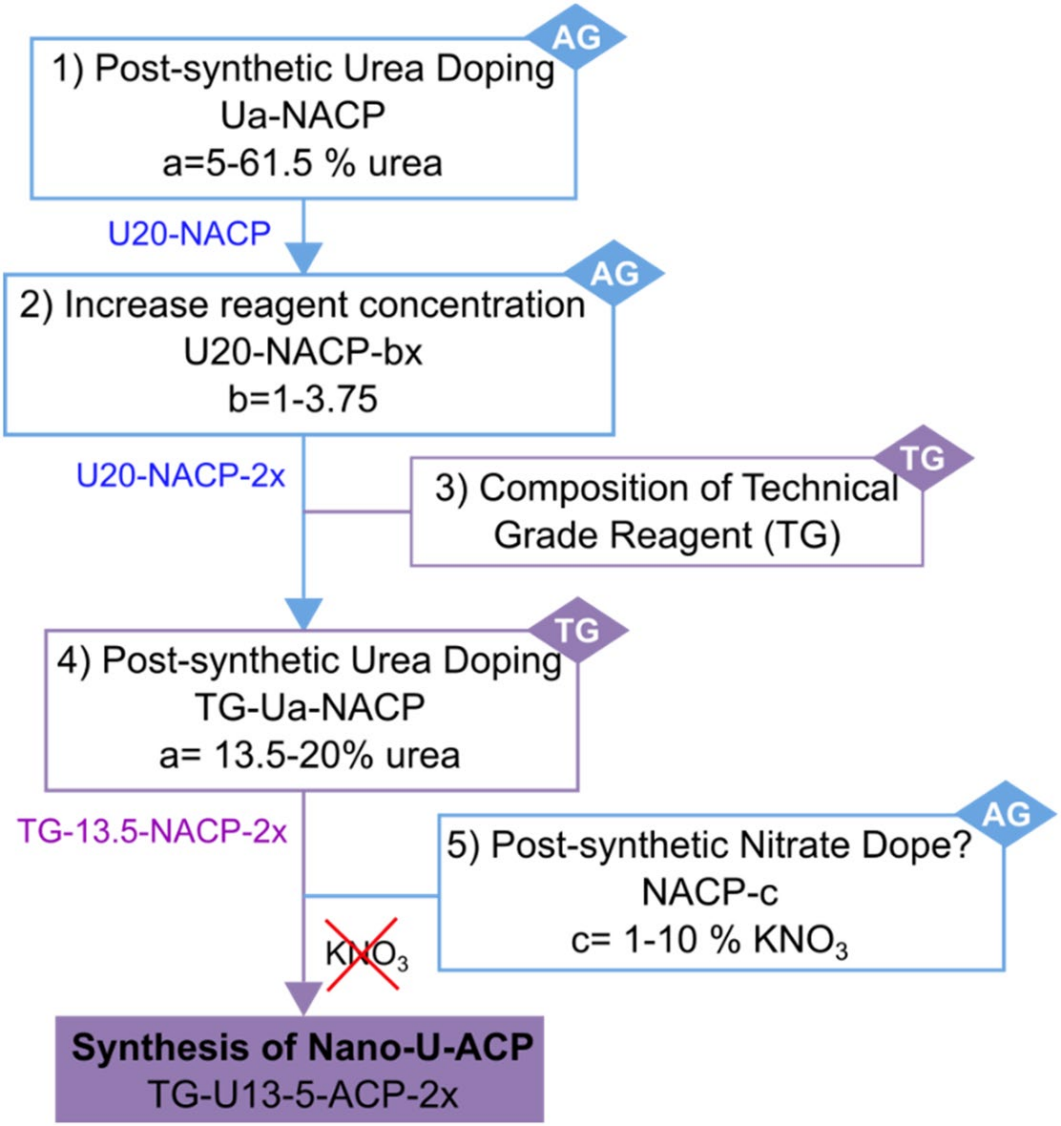
A flow-chart illustrating the steps which enabled the optimization of the PSM process and the sample labelling. AG: analytical grade reagents; TG: technical grade reagents. Note that the weight percentages refer to the synthetic conditions used, and not to the amount of urea or nitrate ion incorporation.

**Figure 1:**
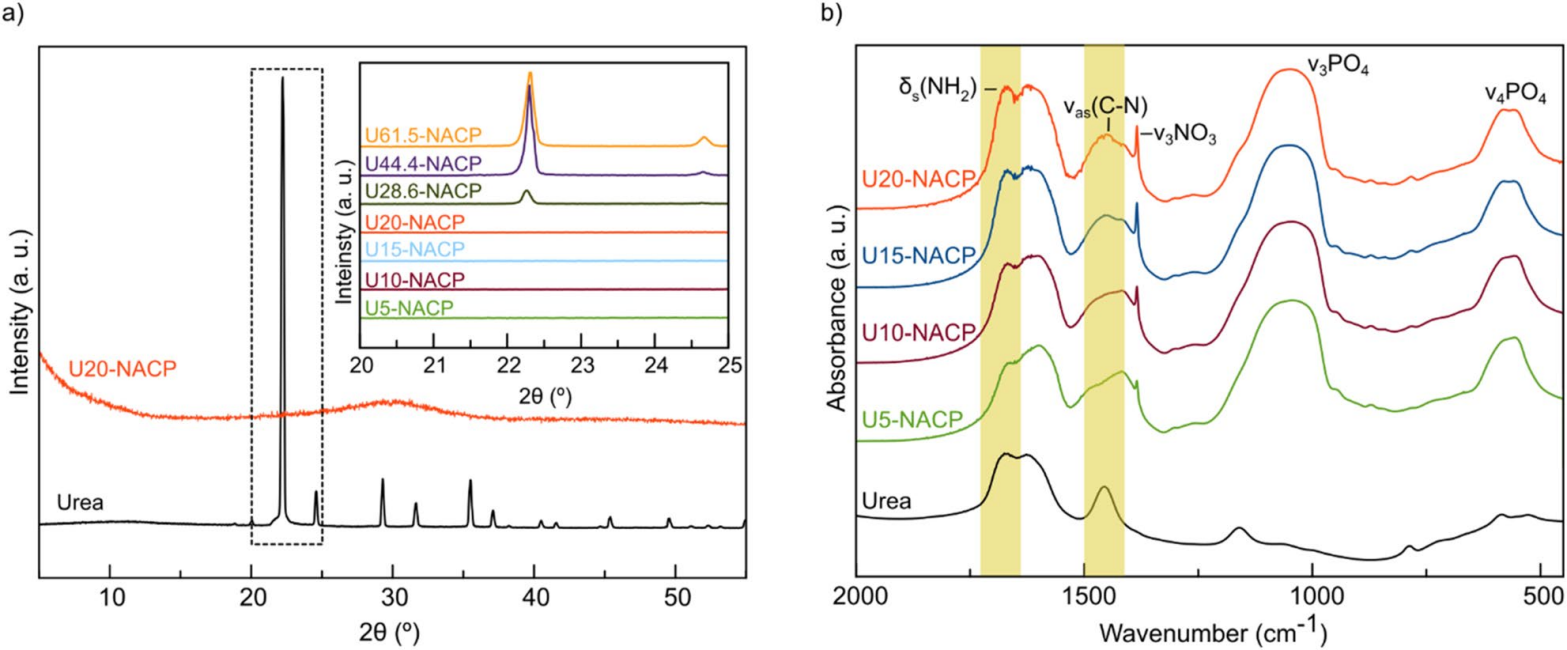
(**a**) X-ray powder diffractograms of pure crystalline urea (black line) and U-20-NACP material (orange line). Inset: X-ray diffractograms (2θ from 20° to 25°) of U-doped materials. (**b**) FT-IR spectra of pure crystalline urea (black line) and U5-NACP, U10-NACP, U15-NACP and U20-NACP materials. The absorption bands of urea are highlighted in yellow.

Scheme 2 illustrates the workflow related to the PSM design and shows the labeling of the different samples. With reference to step 1, the materials labelled as U*a*-NACP (U = urea, *a* = mass fraction of urea ranging between 5 and 61.5 wt% of the NACP NPs, Table 1, with N indicating the nitrate doping of ACP), were initially characterized by X-ray powder diffraction (XRPD). As shown in Fig. 1, urea concentrations higher than 0.04 M (corresponding to a mass fraction of urea higher than 20%, see Table 1) result in the co-precipitation of segregated urea crystal phase within the otherwise amorphous ACP materials. At variance, no characteristic XRPD peaks of crystalline urea are observed when the mass fraction of urea is not above 20%, as in the U5-NACP, U10-NACP, U15-NACP and U20-NACP samples. XRPD patterns of these materials only show the typical pattern of ACP^28^.

**Table 1:** Synthetic conditions for the materials prepared during the optimization of the amount of urea adsorbed in the NPs. In all cases, solution A contained Na_3_(Citrate) (0.20 M) and Ca(NO_3_)_2_ (0.20 M); solution B contained K_2_HPO_4_ (0.12 M), Na_2_CO_3_ (0.10 M) and KNO_3_ (0.20 M). The concentration of urea is calculated as amount of urea added per total volume of the reaction. The total nitrogen content (wt%) from elemental analysis is provided for fully amorphous samples.

Sample	Urea added (M)	Total N wt%
U5-NACP	0.01	2.40
U10-NACP	0.02	4.24
U15-NACP	0.03	6.31
U20-NACP	0.04	8.13
U28.6-NACP	0.07	Crystalline urea
U44.4-NACP	0.13	Crystalline urea
U61.5-NACP	0.27	Crystalline urea

Noteworthy, the freeze-drying of urea solutions, in the absence of ACP NPs, resulted in the formation of crystalline urea, discarding the potential precipitation of *amorphous urea* (see Methods)*.* This evidence certifies that urea molecules in U5-NACP, U10-NACP, U15-NACP and U20-NACP samples are either adsorbed on the surface of the NPs, incorporated in the amorphous phosphate phase, or both.

These entirely amorphous materials were also analyzed by infrared spectroscopy. FTIR spectra of all samples show the characteristic broad bands of the vibrational modes of phosphate typically found in ACP (500–630 cm^−1^ and 1000–1200 cm^−1^) along with vibrational modes of carbonate (1400–1580 cm^−1^) and citrate ions (1400–1590 cm^−1^) and water (1650 cm^−1^)^28,34^ (Fig. 1b). The sharp band at 1385 cm^−1^, assigned to the antisymmetric stretching v_3_ mode of NO_3_^−^_,_ confirms the functionalization with nitrate ions^35^. Likewise, the characteristic bands of urea^36^ (*i.e.* δ_s_(NH_2_) + νCO vibrations at ≈ 1682 cm^−1^, νCO + δ_s_(NH_2_) vibrations at ≈ 1600 cm^−1^, δ_as_(NH_2_) vibration at ≈ 1630 cm^−1^ and ν_as_(C-N) stretching at ≈ 1465 cm^−1^), observed in the spectra of all functionalized materials, certify the success of the doping process carried out by the novel procedure.

As expected, the intensity of the bands associated to urea increases with the amount of urea employed during the doping. This trend is confirmed by elemental analysis, showing a nitrogen content for U5-NACP, U10-NACP, U15-NACP and U20-NACP of 2.4, 4.24, 6.31 and 8.13 wt%, respectively (Table 1). Noteworthy, while a material with a N-content of 2.8 wt% was previously obtained through the one-pot doping approach by adding 0.66 M of urea and 0.3 M of nitrate ions to the reaction^27^, a similar N-payload is here reached through the post-synthetic doping using the same amount of nitrate (0.3 M of [NO_3_^−^]), and 60 times less urea (0.01 M of added urea). More interestingly, the nitrogen content can be nearly triplicated by using the PSM approach (8.1 to 2.8 wt% N), by adding 16.5 times less urea and the same amount of nitrate. This evidence brings to light the high efficiency of the newly proposed approach to doping.

Precipitation of urea/HAp nanocomposites were recently reported by Kottegoda et al*.,*^15^ who also used addition of urea solutions during synthesis and, as done in our work, avoided subsequent washing. However, the calcium phosphate phase in Kottegoda’s work was a crystalline HAp material and a much higher urea/HAp ratio than in our method was used (6:1) therein. Kottegoda’s resulting material exhibited *co-precipitated* crystalline urea (X-ray evidence), leading to a conglomerate, *i.e.* a physical mixture of microcrystalline urea together with a nanostructured HAp powder carrying adsorbed urea molecules. On the contrary, the powders proposed in the present work are based on small NPs of a *fully amorphous* calcium phosphate. In comparison to crystalline HAp, ACP shows higher solubility^20^, surface reactivity, short-range order and a higher capability of ACP to incorporate (or adsorb) dopants, enabling superior nitrogen payloads^27^. Moreover, ACP can be obtained at very short precipitation times and at room temperature in comparison to (nano)crystalline CaP which require longer maturation times and and/or gentle heating. Accordingly, U*a*-NACP and U*a*-ACP materials (later discussed) are considerably distinct from the reported urea/HAp composites^15^. Encouraged by the elevated level of functionalization obtained by means of post synthetic doping process (urea content up to 20 wt%) while simultaneously avoiding the co-precipitation of segregated crystalline urea, a new screening was performed to maximize the mass production of this reaction (Step 2 of Scheme 2). To this goal, different doped materials were prepared by increasing the concentration of reagents and keeping the proportion of added urea constant at 20 wt% (Table 2). The space–time yield (STY) of the reaction, measuring the efficiency in continuous industrial processes, and the purity of the amorphous phase were the parameters used to optimize the process, though being it performed in batch precipitation mode.

**Table 2:** Synthetic conditions for the materials prepared during the optimization of the amount of urea adsorbed in the NPs. In all cases, solution A contained Na_3_(Citrate) (0.20 M) and Ca(NO_3_)_2_ (0.20 M); solution B contained K_2_HPO_4_ (0.12 M), Na_2_CO_3_ (0.10 M) and KNO_3_ (0.20 M). The concentration of urea is calculated as amount of urea added per total volume of the reaction. The total nitrogen content (wt%) from elemental analysis is provided for fully amorphous samples).

Sample	Solution A (M)		Solution B (M)			Urea (M)^a^
	Na_3_(Citrate)	Ca(NO_3_)_2_	K_2_HPO_4_	Na_2_CO_3_	KNO_3_	
U20-NACP	0.20	0.20	0.12	0.10	0.20	0.04
U20-NACP-2x	0.40	0.40	0.24	0.20	0.40	0.08
U20-NACP-2.5x	0.50	0.50	0.30	0.25	0.5	0.1
U20-NACP-3.75x	0.75	0.75	0.45	0.38	0.75	0.15

^a^ Concentration calculated as amount of urea added per total volume of the reaction.

**Figure 2:**
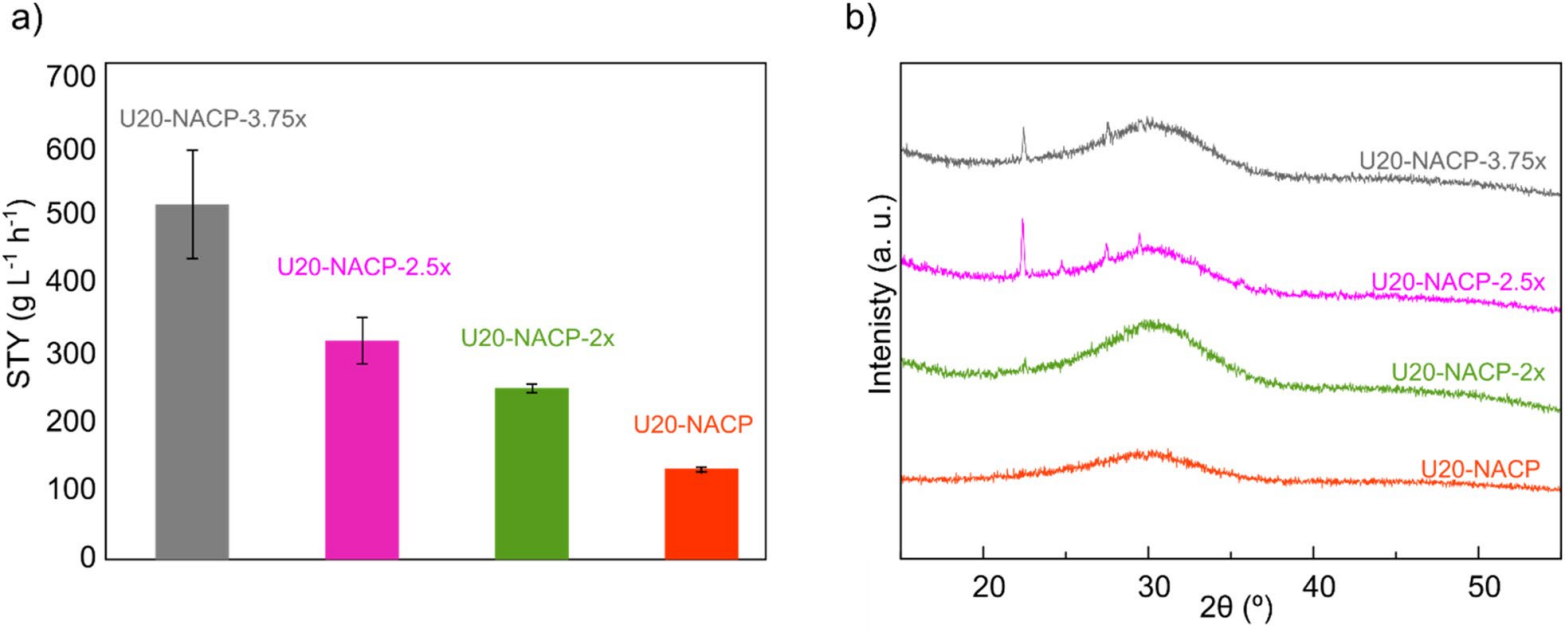
(**a**) Space–time yield of the reaction (STY) calculated for U20-NACP-3.75x, U20-NACP-2.5x, U20-NACP-2 × and U20-NACP. Data are expressed as mean (n = 3) with the corresponding standard error shown as error bars. (**b**) XRPD patterns of the hybrid materials. The characteristic reflections of urea, falling at 2θ = 22.4°, 24.6° and 29.3°, can be appreciated for the materials obtained by increasing the concentration of reagents 2.5 x, or higher than in the preparation of U20-NACP.

As expected, the greater the concentration of reagent the higher the STY of the reaction (Fig. 2a and Table S2). Likewise, FTIR analysis confirms the success of doping by the presence of the characteristic bands of urea in all spectra of the hybrid materials (Figure S1). However, XRPD data show the presence of crystalline urea co-precipitated with the doped amorphous material when the reagent concentration is increased by two times more (or higher) than in U20-NACP (Fig. 2b). Based on this evidence, U20-NACP-2 × was then selected as the optimal material, in terms of urea payload and STY.

### Cost-effective N-doped ACP NPs prepared by using inexpensive reagents

Once we found the best conditions for the preparation and doping of U*a*-NACP (namely the U*20*-NACP), we proved the feasibility of producing similar materials by using TG reagents and tap water, to which the TG-U*a*-NACP label is assigned (Step 4 of Scheme 2). Phase purity or phase composition of TG reagents was assessed by XRPD (step 3 of Scheme 2). Details related to steps 3 and 4 can be found in the Supporting Information (Figures S1-S3 and S5, Tables S1 and S3). Preparation of the TG-U*20*-NACP material having the same grade of functionalization as in the optimized AG-counterpart led to partial co-precipitation of crystalline urea. The reduction of the added urea to a 13.5 wt % level enabled obtaining an entirely amorphous material with a still significant total N payload of 6.8 wt % (Figure S2). Moreover, we noticed that the efficiency of the nitrate doping of the starting NACP was only a 3.42%, being more than 96 wt% lost during the NPs washing process. Attempts to increase the nitrate doping by means of the PSM approach (step 5 of Scheme 2) was not successful either (Figure S3, details in the Supporting Information), resulting in nitrate payloads of less than 2 wt % without co-precipitation of crystalline KNO_3_. These results bring to light the much higher affinity of urea molecules toward ACP NPs than nitrate groups.

**Figure 3:**
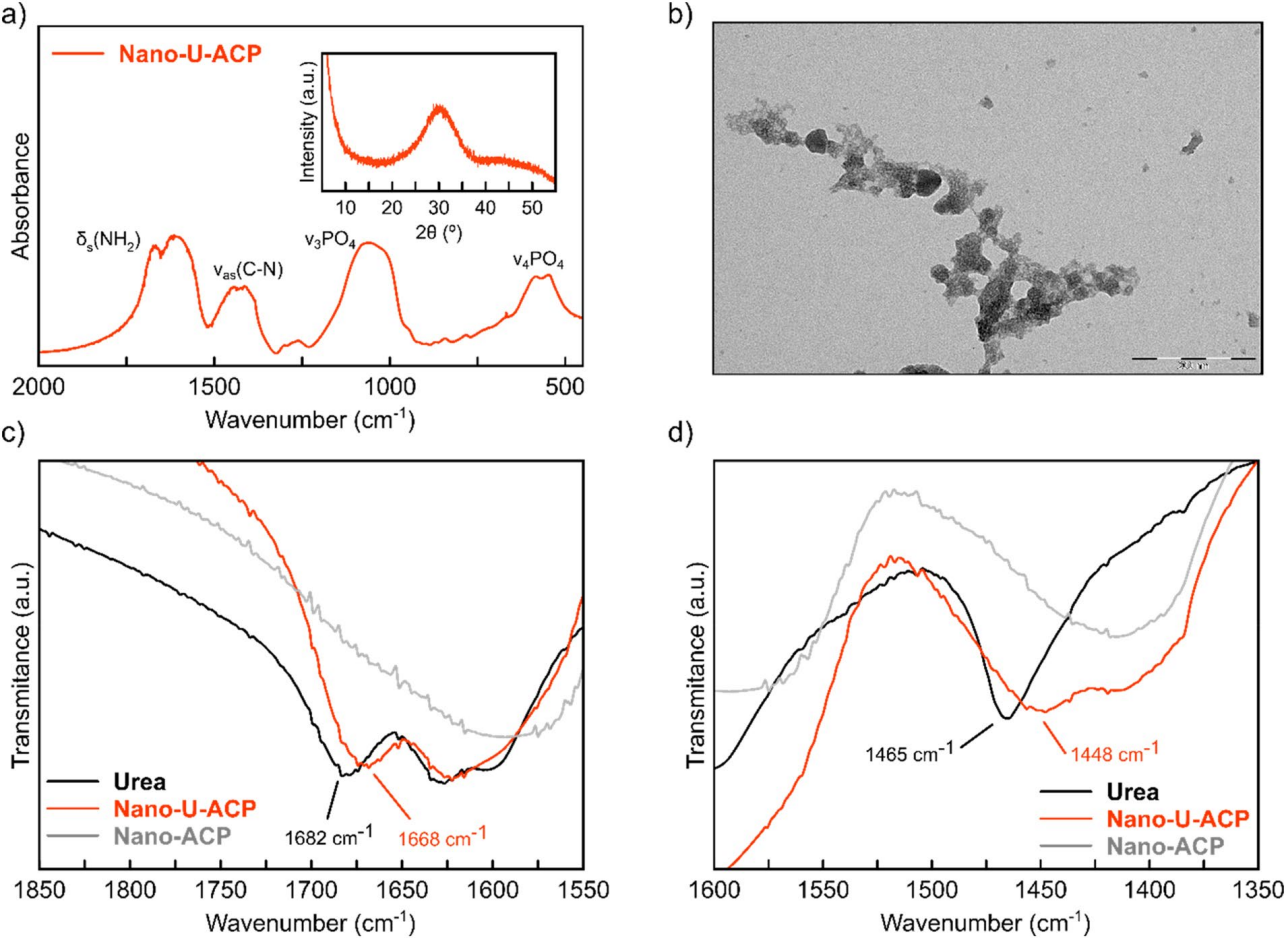
(**a**) FTIR analysis of Nano-U-ACP showing the typical spectrum of ACP as well as the characteristics bands of urea. Inset: X-Ray Powder diffractogram of Nano-U-ACP confirming its pure amorphous nature, (**b**) TEM-image showing Nano-U-ACP NP aggregates with irregular morphology (scale bar = 200 nm). Tiny individual dots with sizes below 10 nm (in line with SAXS results), are also imaged in the rightmost portion; (**c**) and (**d**) FTIR spectra of Urea (black curve), Nano-U-ACP (red curve) and non-functionalized ACP (Nano-ACP, grey curve) in the regions of wavenumber from 1850–1550 cm^−1^ and 1600–1350 cm^−1^, respectively. The shift observed in the vibration mode assigned to the urea for the material Nano-U-ACP and pristine urea are highlighted.

Based on such evidence, we decided to prepare a nitrate-free ACP material doped *only* with urea (the Nano-U*a*-ACP sample), by employing the previously optimized conditions: *i)* TG reagents with doubled (2x) concentration, *ii)* tap water and *iii)* 13.5% mass fraction of urea. In Fig. 3a, XRPD confirms its fully amorphous nature, and FTIR spectroscopy the success of the doping with urea. The N content found by elemental analysis (6.43% ± 0.30) closely matches the expected value (6.30%) and corroborates the successful, and complete, functionalization by urea, while the calcium to phosphate molar ratio estimated by ICP-OES (1.92 ± 0.02) appears rather large, but falls in the wide range typical of amorphous calcium phosphates^37^. Such large value may speak for the possible presence of oxo, hydroxyl or carbonate ions, replacing phosphates within the ACP NPs, or on their surfaces. The TG-U13.5-U*a*-ACP-2 × material, hereafter shortened as Nano-U-ACP (last step of Scheme 2), was then selected as the nanofertilizer for the in vivo tests on cucumber plants growth. As such, it underwent additional physico-chemical characterization that includes TEM and SAXS analysis.

Imaging Nano-U-ACP by TEM indicates the presence of irregularly shaped aggregates (in the 20–40 nm range) of smaller particles (Fig. 3b). Distinguishing the size and shape of the primary particles by TEM was not viable. As only a very small portion of the sample is normally imaged, TEM does not hold a clear statistical robustness. Therefore, we resorted to SAXS analysis, performed on a much larger volume (mm^3^) of the NACP NPs. SAXS data were fitted using a polydisperse ensemble of spherical particles (Figure S4), with number-based average diameter of 6.8 nm and 63% polydispersity, suggesting that the objects imaged by TEM are aggregates of much smaller NPs. FTIR spectroscopy shows that both δ_s_(NH_2_) and ν_as_(C-N) vibration bands are slightly displaced in the Nano-U-ACP material from those measured for crystalline urea (see Fig. 3c,d). These shifts suggest that urea interacts with ACP NPs primarily through its NH_2_ arms, possibly coordinating to the calcium ions on the surface.

### Macronutrient release from cost-effective N-doped ACP NPs

Nutrient release in water was evaluated for Nano-U-ACP that, based on the results earlier discussed, was selected as the optimal TG material concurrently best-performing in terms of urea payload, mass production during synthesis, absence of crystalline components and efficiency of doping. Aqueous suspensions of Nano-U-ACP (1.0 g L^−1^) were prepared and kept at room temperature under stirring. At different times, the supernatant was separated from the solid by centrifugation and the concentration of calcium ions and urea was quantified by ion-selective electrode analysis and the *p-*dimethyl-amino-benzaldehyde colorimetric method^38^, respectively. Given that ACP is mainly formed by calcium and phosphate, the quantitative evaluation of Ca^2+^ ion release from Nano-U-ACP gives information about the solubility of the NPs in water and, indirectly, addresses the amount of phosphate released, toward potential applications of Nano-U-ACP as a simultaneous N and P-fertilizer.

The analysis of the urea release profile confirms the full reversibility of the adsorption process (Figure S5). A sharp *burst effect* is observed during the first 15 min, releasing the 89% of the urea content (123 mg of urea per gram of Nano-U-ACP material). We attribute such burst release to weakly bound urea molecules and/or molecules entrapped between grains. A more gradual release is observed at longer times, reaching the 100% of the urea release after 24 h of suspension, when 138 mg of urea are solubilized. The urea release kinetics profile matched that of the nanofertilizer synthesized through the one-pot preparation, indicating that the same urea-nanoparticle interaction is at work. Thus, the nutrient delivery at longer times showed by both nanomaterials is preferred than the instantaneous release offered by the highly soluble crystalline urea. Interestingly, a comparative test of urea release from the one-pot nanofertilizer and crystalline urea pellets in a low-diffusive medium (mimicking an inert soil) under water irrigation, demonstrated that after 1 h the amount of released urea is 10 times lower when incorporated into the nanofertilizer^27^. A similar amount of reduction is therefore expected for the Nano-U-ACP material in equivalent conditions. Direct comparison of this behavior with systems in which coatings or urease inhibitors have been designed with the specific aim of reducing losses of ammonia from urea fertilizers, would require dedicated experiments. However, analogous tests have been recently reported, which were performed in parallel with sandy loam columns but under static conditions (*i.e.* without a steady water flow). These tests showed that prilled urea granules are relatively rapidly dissolved (in less than 3 days), while their polymer-coated forms were, at least in part, recovered after 2 weeks or so^39^. Thus, though being these urea-based fertilizers studied under different conditions than ours, an approximate tenfold reduction of urea dissolution similar to that observed in Nano-U-ACP materials was achieved by coating. Thus, the two (one-pot synthesis and PSM) strategies offer similar advantages in terms of urea release kinetics, seemingly comparable to the reported case of reduced urea dissolution in polymer-coated form, with the manifest additional (environmental and processing) benefits of the PSM *vs* the one-pot materials discussed in the previous section.

### Compared performances of other phosphate-based fertilizers

Nano-U-ACP frees only the 9.4 wt % of the whole calcium content after 72 h of incubation (see Figure S5), revealing that the calcium ions release process is governed by the progressive degradation of the ACP NPs, which are only partially soluble in water. Differently from nanocrystalline HAp, which has been recently proposed as slow-release P-fertilizer^12,14,18^. ACP shows a higher solubility in neutral or slightly acidic media^22^, enabling a higher delivery of the Ca and P nutrients to the plants, particularly if foliar (where pH is typically below 6) or soilless conditions (where acidity is regulated by organic acid exudation) are employed.

Conventional N,P fertilizers, such as monoammonium phosphate (MAP) and diammonium phosphate (DAP), contain a significantly larger nitrogen content than Nano-U-ACP, reaching the 12.2 and 21.2 wt% N-levels, respectively. However, MAP and DAP are highly soluble salts. More similar to Nano-U-ACP material is the mineral struvite, NH_4_MgPO_4_⋅6H_2_O, in which N amounts to 5.7 wt%. Struvite has also been proposed as an alternative N,P fertilizer^40^, with the additional advantage that it can be recovered by easily crystallization from wastewaters^41^. Details concerning Mg *vs* Ca orthophosphates and their applications (out of the scope of the present work) have been recently reviewed^42^. Struvite is a poorly soluble material, with r.t. solubility in water of only 169 mg L^−1^, but is characterized by rapid (partial) dissolution, which reaches a plateau within 10 min^43^. Recent experiments performed in the presence of organic acids exuded from plant roots demonstrated that struvite dissolution is terminated only after two weeks, nearly independently of the pH value (within the 5.5–8.0 range)^44^. These studies were performed on micrometric crystals, with a specific focus on the agronomic effectiveness of struvite addition in a variety of soils and at different pH, granulation and grinding conditions^45^. Nanosized struvite prepared using wet chemical precipitation methods is yet poorly investigated and specific studies on the dissolution properties are not available. Despite that our Nano-U-ACP material shows a comparable solubility to struvite (computed from the data shown in Figure S5 to be *ca.* 100 mg L^−1^), the gradual and partial Ca^2+^ and phosphate ions release, taking place in several days (Figure S5), indicates a slow NPs dissolution. Finally, both struvite and nanoACP-based fertilizers have a low salt index and thus are of low danger of burning plant roots.

### Test in plants

**Figure 4:**
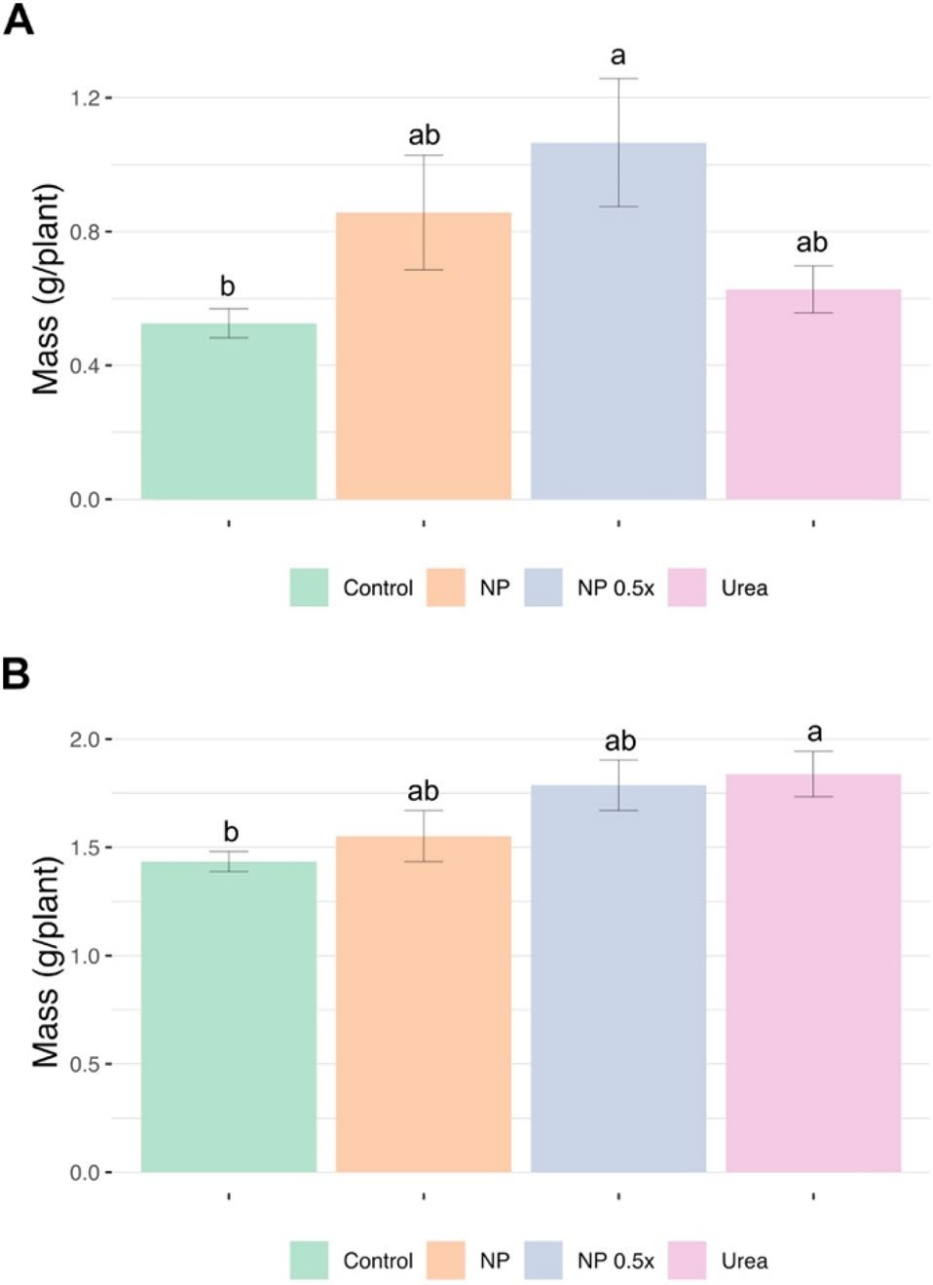
Fresh biomass of roots (**a**) and shoots (**b**) in cucumber plants at 7 days after the treatments. Data are means (± standard error) of five independent biological replicates, (*n* = 5). The statistical significance was determined using one-way ANOVA with Tukey post hoc test (P < 0.05). Different letters indicate significant differences among the treatments.

To unravel the possible influence of NP treatments on plant growth, a fertilization experiment was undertaken using hydroponically grown *Cucumis sativus L.* as model. After germination, plants were grown in a full nutrient solution for 7 days, followed by further 7 days N starvation. Plants were afterward either left in N starvation condition (control) or treated with Nano-U-ACP (NP and NP 0.5 × counting for 1 mM and 0.5 mM content of urea, respectively) and 1 mM urea. The NP 0.5 × treatment was inspired by previous trials on wheat plants demonstrating that those treated with ACP NPs at reduced nitrogen dosages (by 40%) showed unaltered yields and quality in comparison to positive control plants (treated with the minimum N dosages to obtain the highest values of yield and quality in fields)^29^. The analyses performed on the cucumber plants fresh weight showed that both NP and Urea-treated samples increased the root biomass allocation as compared to control samples, although not significantly (Fig. 4a). On the other hand, plants treated with NP 0.5 × significantly enhanced the accumulation of root biomass with respect to control samples (Fig. 4a). Conversely, concerning shoots, the higher biomass was reached by seedlings treated with 1 mM urea in comparison with control plants. Nevertheless, the shoot fresh weight recorded in cucumber plants treated with either NP or NP 0.5 × was not statistically different as compared to the values obtained in urea-supplied plants (Fig. 4b).

Our findings confirm previous observations according to which increasing rates of urea fertilization (up to 3 mM) can induce an increase in the allocation of biomass in plants^46^. Nonetheless, the results reporting a slightly higher growth of the root system in plants supplied with NP 0.5 × as compared to the other samples, might suggest possible inhibitory effects of the higher fertilizer dosages on cucumber (Fig. 4a). Plants are known to be able to directly take up urea as N source through the activity of a specific transporter encoded by different gene *DUR3*, firstly discovered in *Arabidopsis thaliana* and afterwards also characterized in *Zea mays*^46,47^. However, in natural environments, after the application, urea can undergo an enzymatic hydrolysis performed by the bacterial urease enzyme, leading to the release of ammonium^48^. Considering that the hydroponic culture adopted in the present experiment does not feature sterile conditions, we cannot exclude that either endophytic or root-associated microorganism might have catalyzed hydrolysis of urea to ammonium. In this sense, it has been widely established that ammonium as sole source of N for the mineral nutrition of agricultural plants can have detrimental effects on the growth of crops and on the accumulation of both root and shoot biomass^48–50^.

**Figure 5:**
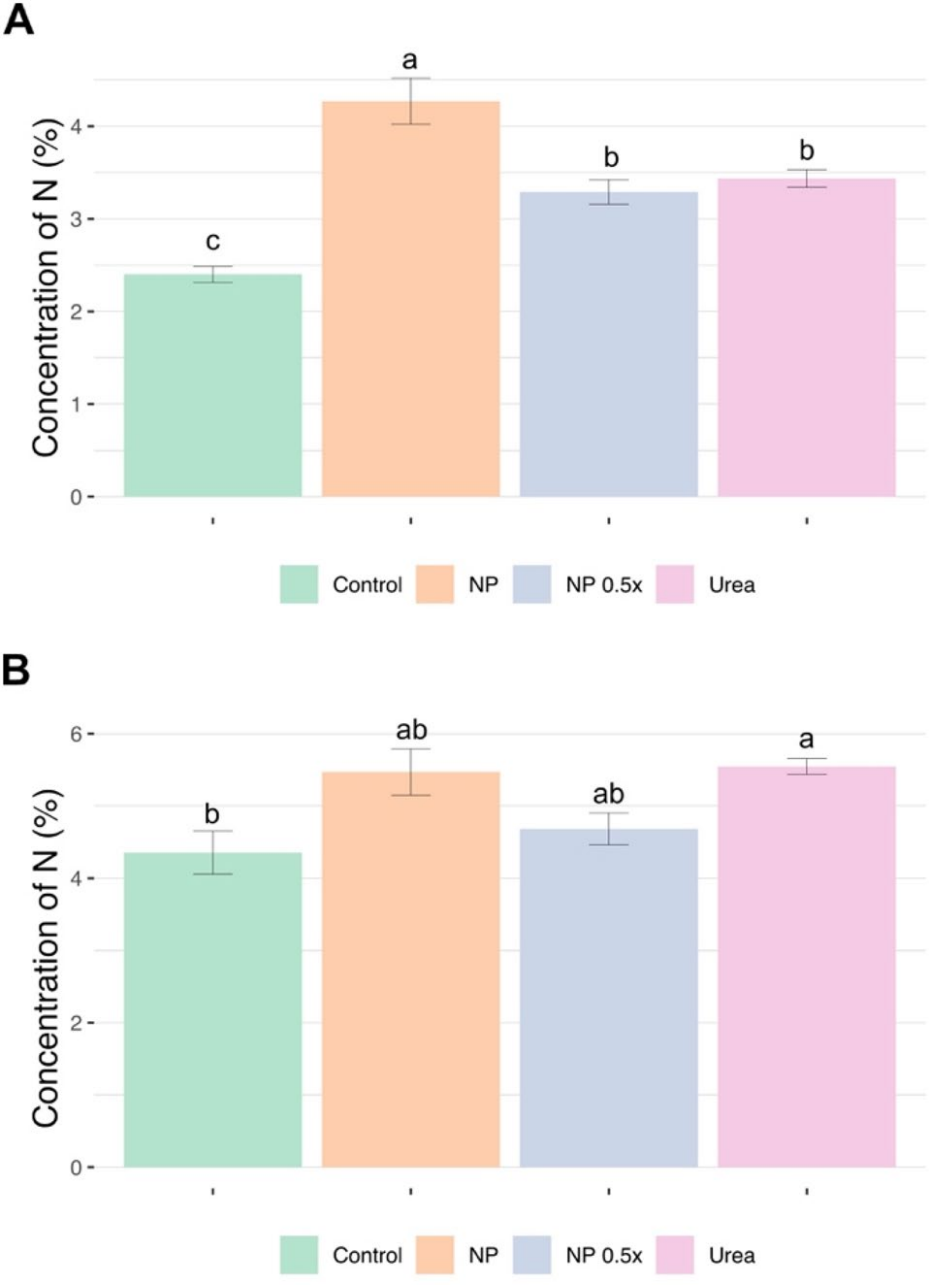
Total N concentration determined in roots (**a**) and shoots (**b**) of cucumber plants at 7 days after the treatments. Statistical analysis has been carried out as specified in Fig. 4.

The IRMS analyses of the present work carried out on roots showed that, 7 days after the treatments, plants treated with full-strength Nano-U-ACP (NP) show the highest level of N concentration (Fig. 5a). At the same time, plants supplied with NP 0.5 × and 1 mM urea displayed a lower N concentration at root levels as compared to NP samples, yet higher with respect to control samples and equivalent among them (Fig. 5a). At shoot level, the highest significative concentration of N is reported in seedlings treated with 1 mM urea as compared to control plants (Fig. 5b); nonetheless, the concentration of N detected in shoots of NP and NP 0.5 × treated samples was not significantly different with respect to that obtained in 1 mM urea-supplied plants. Summarizing, despite the supplementation with NP 0.5 × was adopted to convey a total N amount that is half of that supplied with 1 mM urea, the results hereby presented demonstrated that root and shoot biomasses produced by 0.5 × NP-treated cucumber plants are similar to those accumulated by 1 mM urea supplied plants; in addition, the concentration of N in both shoot and root was comparable. Importantly, previous reports have also established that plants supplemented with either 0.5 mM or 1 mM urea did not show any significant alteration in the phenotypic traits related to the growth; on the other hand, the 1 mM-treated plants displayed a higher urea uptake rate as compared to the 0.5 mM-treated ones^51^. These observations therefore suggest that NP 0.5 × could represent a more efficient fertilization treatment as compared to 1 mM urea. Indeed, cucumber plants fed with NP 0.5 × displayed a NUE of 0.686 ± 0.021, that was statistically higher (p < 0.05) than the NUE coefficient determined for NP (0.478 ± 0.011) and for 1 mM Urea (0.492 ± 0.011).

### Sustainability issues

The development of a material that is suitably engineered for application in agriculture to efficiently deliver nutrients to plants presents multiple sustainability aspects. As for the sustainability of the chemical process, we highlight that the entire protocol here proposed, from the preparation of ACP NPs (intrinsically serving as slow-release P fertilizers) to the urea-based “post-synthetic modification”, has been performed in water, avoiding any hazardous solvent. The PSM of ACP NPs was designed to ensure that the amount of urea added is fully inserted in the final product, increasing the efficient use of starting materials. The significant increase (ca. 3 ×) of the overall N-content, if compared to the one-pot doping approach of ACP NPs, speaks in favor of the atom economy, an important concept of green chemistry philosophy, and one of the most widely used metrics for measuring the "greenness" of a synthetic process.

By developing a synthetic procedure starting from widely available and cheaper commodities (impure reactants and polyphasic mixtures), we provide evidence (in Table S4) of a viable scale-up for industrial level production, susceptible to further optimization, opening the way to economic sustainability of the N,P nanofertilizer holding a high-technology level and with N content appropriate for real applications. Indeed, a ca. tenfold decrease of the reagent cost (per gram of N embedded in the NPs) was obtained (Table S4), which we consider a remarkable step toward cost abatement.

From the agronomic point of view, the case of study here presented in hydroponic cultivation with usage of a N concentration provided by Nano-U-ACP, that is half than in conventional fertilization treatments, resulted in the experimentally observed increase of the NUE value by nearly 20%. In this regard, two beneficial aspects can be highlighted: the potential impact of soilless cultivation in terms of urban and vertical farming, particularly where arable lands are scarce (as briefly discussed in the Introduction section), and the advances in search for possible alternatives to pure urea fertilization. Indeed, despite that urea has the lowest price on the market and remains the most important N fertilizer, considering the reported impact of its production and usage on climate change issues, a special care in urea management is highly recommended and losses to the environment should be strictly avoided. Compared to crystalline urea, previous tests in low-diffusive media suggest that urea release from Nano-U-ACP can be slowed down by a factor of ten when adsorbed to the NPs surface^27^. This reduction is in line with the values observed for commercial polymer-coated urea granules^39^. It is worthwhile mentioning that reduced amounts of our N,P-nanofertilizer compared to conventional fertilization protocols were effective also in soil and foliar applications, as witnessed by previous tests with the one-pot N,P nanofertilizer (having lower N content) on wheat and grapes^27,30^. Further advances in this respect rely on applying nanotechnology concepts to innovative plant nutrient supplies, toward preservation of non-renewable phosphate ores. All these findings make the product and the protocol herein proposed environmentally friendly and, to some extent, economically sustainable.

## Conclusions

Based on the promising features showed by Amorphous Calcium Phosphate (ACP) as a platform for multinutrient nanofertilizers, we have developed an efficient approach to dope ACP with urea-based N-macronutrients. In contrast to a one-pot functionalization, the post-synthetic modification approach herein proposed offers higher N-payloads (up to 8.1 wt %, from the initial 2.8 wt % level), a much higher efficiency (namely, use of 16.5 times less urea) and a doubled space–time yield.

Towards the real implantation of N-doped ACP as a nanofertilizer, we have proved the feasibility of producing Urea-doped ACP materials (Nano-U-ACP) by using inexpensive, technical-grade reagents and tap water. Given the high surface reactivity of ACP and the elevated affinity of urea molecules to Ca^2+^ ions, Nano-U-ACP showed a still high payload (N content of 6.43 wt %). Noteworthy, all the urea used in the process was incorporated in the material avoiding unwanted losses and, therefore, reducing both the economic cost and the environmental risks of the process. Further coating with biocompatible, slowly dissolving polymers can be envisaged toward a more sustained release of urea, at the expenses of a more complex and costly synthetic work-up.

The fertilization tests carried out on hydroponically grown *Cucumis sativus L* as a plant model have demonstrated the higher nutrient use efficiency showed by Nano-U-ACP (NUE 69%) than its conventional fertilizer counterpart (urea, NUE 49%). The use of Nano-U-ACP with a 50% reduced N content of urea resulted in similar root and shoot biomass, as well as statistically equivalent N content in both of them.

## Methods

### Materials

The analytical grade reagents, namely calcium nitrate tetrahydrate (CaNO_3_·4H_2_O, ≥ 99.0% pure, Bioxtra), sodium citrate tribasic dihydrate (Na_3_(citrate)∙2H_2_O, ≥ 99.0% pure, urea (pellets, ≥ 99.5%, ReagentPlus®), anhydrous dipotassium hydrogenphosphate (K_2_HPO_4_, ≥ 99.0% pure), sodium carbonate (Na_2_CO_3_, ≥ 99.0% pure, Bioxtra) and potassium nitrate (KNO_3_, ≥ 99.0%), were purchased from Sigma Aldrich. The technical grade reagents were purchased from on-line distributors: calcium nitrate, potassium nitrate and urea from AL.Fe (Italy, www.alfenatura.com), dipotassium hydrogenphosphate from MyProtein (UK, www.myprotein.com), sodium citrate dihydrate from Algin-Chemie (Germany, www.algin-chemie.de) and sodium carbonate from buXtrade (Germany, buxtrade.de).

### Material characterization

X-ray Powder diffraction (XRPD) data of the ACP materials prepared during the several screening experiments were collected on a Rigaku Miniflex 300 diffractometer using Cu Kα radiation (λ = 1.5418 Å), from 5° to 55° (2θ) with a step size of 0.02° and scanning rate of 1.0° min^−1^. XRPD data of the Nano-U-ACP labelled material (selected for the in vivo tests, vide infra) and the technical grade reagents were collected on a Bruker AXS D8 Advance diffractometer using Cu Kα radiation (λ = 1.5418 Å), from 5° to 55° (2θ) and 5° to 85°, respectively, with a step size of 0.02° and scanning rate of 1.0 s step^−1^. Elemental (C,H,N) analyses were obtained with a Perkin Elmer 2400 series II instrument from Centre of Scientific Instrumentation of the University of Granada (CIC-UGR). Transmission electron microscopy (TEM) images were collected with a LIBRA 120 PLUS (Carl Zeiss SMT) operating at 120 kV (CIC-UGR). The materials were ultrasonically dispersed in ethanol and then few drops of the slurry were deposited on 200 mesh copper grids covered with thin amorphous carbon films. Fourier transform infrared (FTIR) spectra were collected on a Bruker Tensor 27 spectrometer. 2 mg of the sample were mixed with 150 mg of KBr and pressed by a hydraulic press (Specac, 2 tons) to form a pill. FTIR spectra were collected with a spectral resolution of 2 cm^−1^ by accumulating 32 scans in the 4000–450 cm^−1^ range. In the case of Urea, Nano-ACP and Nano-U-ACP labelled samples (vide infra), FTIR spectra were collected by accumulating 100 scans in the 4000–450 cm^−1^ range. Small-angle X-ray scattering (SAXS) data were collected at Aarhus University (Denmark), using an in-house SAXS instrument equipped with a rotating Cu anode source, a side-by-side Montel multilayer mirrors, a collimating system and a Vantec 500 (Bruker AXS) detector. SAXS analysis was performed to derive size and morphology of the ACP NPs. (Supplementary Text S3). Details on the instrumental setup, sample preparation, data collection and modelling, performed by the DSE method implemented in the DebUsSy code^52^ are provided in the Supporting Information (Supplementary Text S4). Worthy of note, the morphological results derived by SAXS analysis, based on a large ensemble of nanoparticles (> 10^6^) which cannot be imaged by TEM, holds a higher statistical robustness.

### Synthesis of Ua-NACP samples

A PSM of Nitrated-Amorphous Calcium Phosphate (NACP) NPs was performed to evaluate the viability of increasing their urea content in comparison to a previously adopted one-pot synthesis. The preparation of the materials was performed by chemical precipitation with analytical grade (AG) reagents, following a similar procedure to that previously reported for the one-pot method^27^, except that urea was added after the NPs were precipitated and washed (see Scheme 1, later discussed). In a typical preparation, two aqueous solutions (1:1 v/v, 5 mL total) of (i) 0.2 M Ca(NO_3_)_2_⋅4H_2_O, 0.2 M Na_3_(citrate)⋅2H_2_O (ii) 0.1 M Na_2_CO_3_, 0.12 M K_2_HPO_4_, 0.2 M KNO_3_ were mixed at room temperature. The mixture was introduced in a glass bottle sealed with a screw cap (Duran) and heated at 37 °C for 5 min. The resulting solids were collected by centrifugation (10 min, 4500 rpm, room temperature) and washed with water (2 × 10 mL). 1 ml of urea solution, with variable concentrations ranging from 2.6 g L^−1^ to 80 g L^−1^ (corresponding to 0.01—0.27 M per total volume of the reaction, see Table 1), was mixed with the slurry and freeze-dried. These materials are labelled as Ua-NACP (Step 1 of Scheme 2), where a is the nominal mass fraction of urea admixed to the NPs, ranging between 5 and 61.5 wt% of the initial NACP NPs (Table 1). The maximum amount of urea fully adsorbed in the particle surface, namely without co-precipitation of crystalline urea (easily detectable by X-ray powder diffraction), was found to be close to 20 wt%. Precipitation of an *amorphous urea* phase in the inter-grain voids upon freeze-drying aqueous ACP NPs suspensions was considered and eventually discarded. *Amorphous urea* is reported to form either by physical vapor deposition at 84 K^53^ or in supercritical CO_2_ conditions (at pressures above 73 atm)^54^, that is in conditions very far from conventional laboratory ones. Computational modeling indicated that transient urea clusters form during crystallization in acetonitrile or in dissolution processes with nearly null lifetimes^55^. Hence, the hypothesis of an *amorphous urea* phase formed in the synthetic conditions of the present work was abandoned in favor of urea molecules adsorbed at the particles surface or trapped in between. A control experiment was also performed. 1 mL of an aqueous solution of urea (10.4 g L^−1^), paralleling the conditions of the original urea-doped ACP NPs preparation (but in absence of NPs) was freeze-dried. The XRPD pattern of the resulting highly crystalline precipitate is shown in Figure S6.

### Synthesis of U20-NACP-bx samples

Once the amount of doping agent (urea in non-crystalline form) was optimized, a new screening was performed to maximize the space–time yield of the reaction (STY), defined as the mass of product obtained per unit volume of reaction mixture per hour [g L^−1^ h^−1^]. To this aim, several materials were prepared by following the same procedure as above with 20% as mass fraction of urea, except that the concentration of the reagents was scaled up x times. Corresponding samples are labelled as U20-NACP-bx, with b = 1.0, 2.0, 2.5 and 3.75 (Step 2 of Scheme 2). It was observed that values of b > 2 resulted in the co-precipitation of crystalline urea. Therefore, 2 × was selected as the optimum scale factor and the U20-NACP-2 × as the reference sample.

### Assessment of the phase composition of technical grade reagents by XRPD

Full details of the compositional and crystal phase analysis of TG reagents are reported in the Supporting Information file (Figures S1, S2 and S3 and Supplementary Text S1).

### Synthesis of NACP and post-synthetic urea-doping – TG reagents

Samples labelled TG-U20-NACP, TG-U*15*-NACP and TG-U*13.5*-NACP (with urea mass fraction of 20%, 15% and 13.5%, respectively) were prepared by following the same procedure than in U*a*-NACP-2 × material, except that TG reagents and tap water (supplied by Acsm Agam Reti Gas Acqua SpA, with a standard certificate of analysis reported in Table S1) were used (Step 4 of Scheme 2). Additional experimental details are reported in Supplementary Text S2.

### Post-synthetic nitrate-doping of ACP

Given the low efficiency of nitrate-doping by means of the one-pot process, a screening was also performed to post-synthetically functionalize ACP materials with nitrate ions, aiming at inserting an additional N source in the form of a non-crystalline material through a process reminiscent of urea functionalization discussed above. Using AG reagents, two aqueous solutions (1:1 v/v, 5 mL total) of (i) 0.2 M Ca(NO_3_)_2_⋅2H_2_O, 0.2 M + Na_3_(citrate)⋅2H_2_O. and (ii) 0.1 M Na_2_CO_3_ + 0.12 M K_2_HPO_4_ were mixed at room temperature. The mixture was introduced in a glass bottle sealed with a screw cap (Duran) and heated at 37 °C for 5 min. The resulting solids were collected by centrifugation (10 min, 4500 rpm, room temperature) and washed with water (2 × 10 mL). 1.0 mL of KNO_3_ solution with concentrations ranging from 2.6 g L^−1^ to 21.4 g L^−1^ were mixed with the slurry and freeze-dried. The resulting samples are labelled as NACP-*c*, *c*: mass fraction of KNO_3_, *c* = 1 − 10%, based on the production of 10 g L^−1^ (Step 5 of Scheme 2).

### Preparation of Nano-U-ACP for in vivo tests

The material was prepared by employing the synthetic conditions optimized through the different screening experiments: *i)* TG reagents with concentration of 2x, *ii)* tap water and *iii)* only urea as post-synthetic dopant, with a mass fraction of 13.5% (vide infra). For this sample, two aqueous solutions (1:1 v/v, 150 mL total) of (i) 0.4 M calcium chloride, 0.4 M Na_3_(citrate)⋅2H_2_O and (ii) 0.2 M Na_2_CO_3_ + 0.24 M K_2_HPO_4_ were prepared and mixed at room temperature. The mixture was introduced in a glass bottle sealed with a Duran screw cap and heated at 37 °C for 5 min. The resulting solids were collected by centrifugation (10 min, 4500 rpm, room temperature) and washed with water (2 × 300 mL). 3 mL of a 160 g L^−1^ urea solution (mass fraction of 13.5%) were mixed with the slurry and freeze-dried. For this material, the Nano-U-ACP label is used throughout the paper, superseding the pertinent TG-U13.5-ACP label (see Scheme 2), being its composition unique. A material without urea doping, labelled Nano-ACP, was also prepared in parallel as a reference material.

### Calcium release from Nano-U-ACP

The amount of Ca^2+^ released from Nano-U-ACP was monitored by using Ca^2+^-selective electrode methodology (Mettler-Toledo). Such release was taken as a measure of nanoparticle decomposition, paralleling the release of phosphate anions, and of occluded carbonates. Prior to each experiment, a calibration curve of Ca^2+^ was performed using CaCl_2_⋅2H_2_O, with three standard solutions containing 10, 100 or 1000 ppm of Ca^2+^, respectively, using KCl 4 M as ionic strength adjustment buffer (ISAB). In a typical experiment, 30 mg of CaP nanoparticles were suspended in 30 mL of water and kept at room temperature under continuous magnetic stirring. At scheduled times (0.25, 1, 3, 24 and 72 h), the suspension was centrifuged (4500 rpm, 10 min) and the supernatant was collected. After adding 2 mL of ISAB, the electrodes were immersed in the solution and the concentration of calcium ions was quantified. All experiments were performed in triplicate.

### Urea release from Nano-U-ACP

The amount of urea released from Nano-U-ACP was spectrophotometrically monitored by means of the *p*-dimethyl-amino-benzaldehyde (P-DMAB) colorimetric method^38^. In a typical experiment, 3 mg of CaP nanoparticles were suspended in 3 mL of water and kept at room temperature. At the scheduled times (0.25, 1, 3, 24 and 72 h), the suspension was centrifuged (4500 rpm, 10 min) and 1.5 mL of the supernatant were added to 1.5 mL of P-DMAB-based reagent color. After 15 min, the concentration of urea was quantified by measuring its absorbance at 445 nm with a Spectroquant Nova photometer (Merck). All experiments were performed in triplicate.

### Plant material and fertilization experiment

Cucumber plants (*Cucumis sativus* L.) were germinated on filter papers, wetted with 0.5 mM CaSO_4_ solution, in a covered tray. After 5 days, seedlings were transferred into 1.5 L pots containing an aerated hydroponic solution composed as follows: Ca(NO_3_)_2_ (2 mM), MgSO_4_ (0.5 mM), K_2_SO_4_ (0.7 mM), KCl (0.1 mM), KH_2_PO_4_ (0.1 mM), H_3_BO_3_ (10 μM), MnSO_4_ (0.5 μM), CuSO_4_ (0.2 μM), ZnSO_4_ (0.5 μM) and (NH_4_)_6_Mo_7_O_24_ (0.01 μM). The Fe-EDTA (80 μM) micronutrient was added at the end, separately for each pot. The solution was replaced regularly every two days. After 7 days of growth in a full nutrient medium, Ca(NO_3_)_2_ was removed from the solution, in order to achieve a period of nitrogen starvation. The Ca^2+^ content was rebalanced adding 0.5 g of bulk CaSO_4_ for each pot. From this point, the nutrient solution was replaced regularly, always maintaining the starvation pattern.

Afterwards, cucumber plants were supplied with different N sources (*i.e.* urea 1 mM, NP and NP 0.5x) defining three types of treatment. The amount of Nano-U-ACP to be added to the NP treatment was calculated in order to supply the same amount of N as compared to the samples added with 1 mM Urea; half of this amount was supplied in the NP 0.5 × treatment. At sampling, that is seven days after the beginning of treatments, shoots and roots were separated, and the fresh biomass recorded. Plant tissues were afterwards dried at 65 °C until constant weight, homogenized and analyzed for the total N content by Isotope Ratio-Mass Spectrometry (IRMS). The fertilization experiments were carried out on five independent biological replicates per each treatment (*i.e.* five independent pots) and each biological replicate was obtained by randomly pooling three cucumber plants. The calculation of Nitrogen Use Efficiency (NUE) coefficient was carried out as previously described by Mauceri et al^56^. Results were obtained by averaging those from five independent replicates; their statistical significance was determined using the one-way ANOVA with Tukey post hoc test (P < 0.05).

## Supplementary Information

Supplementary Information.

## Data Availability

All data generated or analyzed during this study are included in this published article (and its Supplementary Information file).

## References

[1] Freney JR, Simpson JR (1983). Gaseous Loss of Nitrogen from Plant–Soil Systems. Development in Plant and Soil Sciences.

[2] Bremner JM, Blackmer AM (1978). Nitrous oxide: emission from soils during nitrification of fertilizer nitrogen. Science.

[3] Conley DJ (2009). ECOLOGY: controlling eutrophication: nitrogen and phosphorus. Science.

[4] Chislock MF, Zitomer RA, Wilson AE (2013). Eutrophication: causes, consequences, and controls in aquatic ecosystems. Nat. Educ. Knowl..

[5] Naz MY, Sulaiman SA (2017). Attributes of natural and synthetic materials pertaining to slow-release urea coating industry. Rev. Chem. Eng..

[6] Geng J (2015). Synchronized relationships between nitrogen release of controlled release nitrogen fertilizers and nitrogen requirements of cotton. Food Crop. Res..

[7] Chen S (2019). Root-associated microbiomes of wheat under the combined effect of plant development and nitrogen fertilization. Microbiome.

[8] Calabi-Floody M (2018). Smart Fertilizers as a Strategy for Sustainable Agriculture. Advances in Agronomy.

[9] Sega D (2019). FePO4 nanoparticles produced by an industrially scalable continuous-flow method are an available form of P and Fe for cucumber and maize plants. Sci. Rep..

[10] Servin A (2015). A review of the use of engineered nanomaterials to suppress plant disease and enhance crop yield. J. Nanopart. Res..

[11] Kopittke P, Lombi E, Wang P, Schjørring JK, Husted S (2019). Nanomaterials as fertilizers for improving plant mineral nutrition and environmental outcomes. Environ. Sci. Nano.

[12] Liu R, Lal R (2014). Synthetic apatite nanoparticles as a phosphorus fertilizer for soybean (Glycine max). Sci. Rep..

[13] Xiong L, Wang P, Kopittke PM (2018). Tailoring hydroxyapatite nanoparticles to increase their efficiency as phosphorus fertilisers in soils. Geoderma.

[14] Giroto AS, Guimarães GGF, Foschini M, Ribeiro C (2017). Role of slow-release nanocomposite fertilizers on nitrogen and phosphate availability in soil. Sci. Rep..

[15] Kottegoda N (2017). Urea-hydroxyapatite nanohybrids for slow release of nitrogen. ACS Nano.

[16] Marchiol L (2019). Influence of hydroxyapatite nanoparticles on germination and plant metabolism of tomato (*Solanum lycopersicum* L.): preliminary evidence. Agronomy.

[17] Xiong L, Wang P, Hunter MN, Kopittke PM (2018). Bioavailability and movement of hydroxyapatite nanoparticles (HA-NPs) applied as a phosphorus fertiliser in soils. Environ. Sci. Nano.

[18] Giroto AS, Fidélis SC, Ribeiro C (2015). Controlled release from hydroxyapatite nanoparticles incorporated into biodegradable, soluble host matrixes. RSC Adv..

[19] Carmona FJ (2020). The role of nanoparticle structure and morphology in the dissolution kinetics and nutrient release of nitrate-doped calcium phosphate nanofertilizers. Sci. Rep..

[20] Wang L, Nancollas GH (2008). Calcium orthophosphates: crystallization and dissolution. Chem. Rev..

[21] Dorozhkin SV (2007). Calcium orthophosphates. J. Mater. Sci..

[22] Dorozhkin SV, Epple M (2002). Biological and medical significance of calcium phosphates. Angew. Chem. Int. Ed..

[23] Vandecandelaere N, Rey C, Drouet C (2012). Biomimetic apatite-based biomaterials: on the critical impact of synthesis and post-synthesis parameters. J. Mater. Sci. Mater. Med..

[24] Bertolotti F (2021). On the amorphous layer in bone mineral and biomimetic apatite: a combined small- and wide-angle X-ray scattering analysis. Acta Biomater..

[25] Delgado-López JM (2012). Crystallization of bioinspired citrate-functionalized nanoapatite with tailored carbonate content. Acta Biomater..

[26] Delgado-López JM (2014). Crystal size, morphology, and growth mechanism in bio-inspired apatite nanocrystals. Adv. Funct. Mater..

[27] RamírezRodríguez GB (2020). Engineering biomimetic calcium phosphate nanoparticles: a green synthesis of slow-release multinutrient (NPK) nano-fertilizers. ACS Appl. Bio Mater..

[28] Combes C, Rey C (2010). Amorphous calcium phosphates: synthesis, properties and uses in biomaterials. Acta Biomater..

[29] Ramírez-Rodríguez GB (2020). Reducing nitrogen dosage in triticum durum plants with urea-doped nanofertilizers. Nanomaterials.

[30] Pérez-Álvarez EP (2020). Towards a more sustainable viticulture: N-doped calcium phosphate nanoparticles to enhance nitrogen uptake in grapes. J. Sci. Food Agric..

[31] Beacham AM, Vickers LH, Monaghan JM (2019). Vertical farming: a summary of approaches to growing skywards. J. Hortic. Sci. Biotechnol..

[32] Solanki P, Bhargava A, Chhipa H, Jain N, Panwar J, Rai M, Ribeiro C, Mattoso L, Duran N (2015). Nano-fertilizers and their smart delivery system. Nanotechnologies in Food and Agriculture.

[33] Sambo P (2019). Hydroponic solutions for soilless production systems: issues and opportunities in a smart agriculture perspective. Front. Plant Sci..

[34] Stutman JM, Termine JD, Posner AS (1965). Vibrational spectra and structure of phosphate ion in some calcium phosphates. Trans. N. Y. Acad. Sci..

[35] Miller FA, Wilkins CH (1952). Infrared spectra and characteristic frequencies of inorganic ions. Anal. Chem..

[36] Keuleers R, Desseyn HO, Rousseau B, Van Alsenoy C (1999). Vibrational analysis of urea. J. Phys. Chem. A.

[37] Dorozhkin SV (2013). Nanodimensional and nanocrystalline hydroxyapatite and other calcium orthophosphates. Am. J. Biomed. Eng..

[38] Ceriotti G, Spandrio L (1963). A spectrophotometric method for determination of urea. Clin. Chim. Acta.

[39] Campos OR, Mattiello EM, Cantarutti RB, Vergütz L (2018). Nitrogen release from urea with different coatings. J. Sci. Food Agric..

[40] Ricardo GP, López-de-Sá EG, Plaza C (2009). Lettuce response to phosphorus fertilization with struvite recovered from municipal wastewater. HortScience.

[41] Rahman MM (2014). Production of slow release crystal fertilizer from wastewaters through struvite crystallization—a review. Arab. J. Chem..

[42] Nabiyouni M, Brückner T, Zhou H, Gbureck U, Bhaduri SB (2018). Magnesium-based bioceramics in orthopedic applications. Acta Biomater..

[43] Babić-Ivančić V, Kontrec J, Kralj D, Brečević L (2002). Precipitation diagrams of struvite and dissolution kinetics of different struvite morphologies. Croat. Chem. Acta.

[44] Talboys PJ (2016). Struvite: a slow-release fertiliser for sustainable phosphorus management?. Plant Soil.

[45] Degryse F, Baird R, da Silva RC, McLaughlin MJ (2017). Dissolution rate and agronomic effectiveness of struvite fertilizers—effect of soil pH, granulation and base excess. Plant Soil.

[46] Zanin L (2014). Isolation and functional characterization of a high affinity urea transporter from roots of Zea mays. BMC Plant Biol..

[47] Kojima S, Bohner A, Gassert B, Yuan L, von Wirén N (2007). AtDUR3 represents the major transporter for high-affinity urea transport across the plasma membrane of nitrogen-deficient Arabidopsis roots. Plant J..

[48] Watson CJ (1994). Soil properties and the ability of the urease inhibitor N-(n-BUTYL) thiophosphoric triamide (nBTPT) to reduce ammonia volatilization from surface-applied urea. Soil Biol. Biochem..

[49] Na L (2014). Effect of nitrate/ammonium ratios on growth, root morphology and nutrient elements uptake of watermelon (*Citrullus lanatus*) seedlings. J. Plant Nutr..

[50] Meng L (2019). Differential responses of root growth to nutrition with different ammonium/nitrate ratios involve auxin distribution in two tobacco cultivars. J. Integr. Agric..

[51] Zanin L (2015). Transcriptomic analysis highlights reciprocal interactions of urea and nitrate for nitrogen acquisition by maize roots. Plant Cell Physiol..

[52] Cervellino A, Frison R, Bertolotti F, Guagliardi A (2015). DEBUSSY 2.0: the new release of a Debye user system for nanocrystalline and/or disordered materials. J. Appl. Crystallogr..

[53] Clout, A. Studying phase transitions and co-crystallisation in pharmaceutical material (2018). https://discovery.ucl.ac.uk/id/eprint/10044058

[54] Cuadra, I. Preparation of pharmaceutical co-crystals, adducts and composites using supercritical CO_2_ as an antisolvent (2019). https://eprints.ucm.es/id/eprint/56778/1/T41349.pdf

[55] Salvalaglio M, Mazzotti M, Parrinello M (2015). Urea homogeneous nucleation mechanism is solvent dependent. Faraday Discuss..

[56] Mauceri A (2020). Genetic variation in eggplant for nitrogen use efficiency under contrasting NO3-supply. J. Integr. Plant Biol..

