# Supplementary material for: Urea-functionalized amorphous calcium phosphate nanofertilizers: optimizing the synthetic strategy towards environmental sustainability and manufacturing costs
Source: Sci Rep. 2021 Feb 9;11:3419. doi: 10.1038/s41598-021-83048-9 (PMC7873063; doi:10.1038/s41598-021-83048-9)
Supplement: Supplementary file 1 — Supplementary Information. [file 41598_2021_83048_MOESM1_ESM.pdf]

Supporting Information

Urea-functionalized amorphous calcium phosphate nanofertilizers: optimizing the synthetic strategy towards environmental sustainability and manufacturing costs

Francisco J. Carmona,* Gregorio Dal Sasso, Gloria B. Ramírez-Rodríguez, Youry Pii, José Manuel Delgado-López, Antonietta Guagliardi,* and Norberto Masciocchi*

Supplementary Tables S1 – S4

Supplementary Figures S1 – S9

Supplementaty Text S1 – S4

Supplementary Tables

Table S1. Chemical and bacteriological analysis of tap water supplied by Acsm Agam Reti Gas Acqua SpA, Como East Section, where the Chemical Building of the University of Insubria is located.

**Item**	**Unit**	**Avererage value, 2018**	**Avererage value, 2019**	**Limiting value**
Alkalinity	mg/l $CaCO_3$	75.36	72.54	/
Ammonia	mg/l NH_3	0.07	0.02	0.5
Arsenic	µg/l As	3.91	3.85	10
Chlorine dioxide	mg/l ClO_2	0.07	0.05	/
Calcium	mg/l Ca	26.79	27.5	/
Chloride	mg/l Cl	5.14	5.08	250
Hydrogen ion concentration	pH units	7.98	8.16	6.5 – 9.5
Electric conductivity	µS/cm	176.44	188.23	2500
Total Hardness	°F	9.69	9.23	/
Iron	µg/l Fe	23.24	10.62	200
Fluorides	mg/l F	0.08	0.2	1.5
Magnesiium	mg/l Mg	7.08	6	/
Manganese	µg/l Mn	2.33	1.15	50
Nitrates	mg/l NO_3^-	4	4	50
Nitrites	mg/l NO_2^-	0	0.1	0.1
Potassium	mg/l K	2.46	1.15	/
Fixed Residue	mg/l	105.77	139.23	/
Sodium	mg/l Na	7.34	6.31	200
Sulfates	mg/l $SO_4^{=}$	27.17	27.31	250
Coliform Bacteria	MPN/100 ml	0	0	0
Enterococci	MPN/100 ml	0	0	0
Escherichia Coli	MPN/100 ml	0	0	0

Table S2. Space-time yield (STY) calculated during the maximization of the NP production.

	Absolute yield, mg	Volume of reaction, mL	Time of reaction min	STY, g L^{-1} h^{-1}
U20-NACP	55.3	5	5	133
U20-NACP-2x	100.0	5	5	240
U20-NACP-2.5x	120.5	5	5	289
U20-NACP-3.75x	195	5	5	468

Table S3. Chemical formula and purity determined for the technical grade reagents.

	Chemical formula	Purity %
Calcium nitrate	$Ca(NO_3)_2 \cdot 2.807\ H_2O$	100[a]
Sodium citrate	$Na_3(Citrate) \cdot 2H_2O$	100[a]
Urea	$CO(NH_2)_2$	100[a]
Potassium phosphate dibasic	$K_2HPO_4 \cdot 1.475\ H_2O$	100[a]
Sodium carbonate	Na_2CO_3	100[a]
Potassium nitrate	KNO_3	97.1[a,b]

[a] Determined by XRPD, [b] Contaminated by KCl

Table S4. Estimation of costs of production per gram of material and mol of nitrogen for U20-NACP, TG-U13.5-NACP, Nano-U-ACP and conventional nitrogen fertilizers source like diammonium phosphate and monoammonium phosphate. This table only relates to the cost of the raw materials, and does not contain costs of labor, equipment, maintenance and managing, and, not to be neglected, environmental ones. While still far from the competing costs of conventional fertilizers, one can easily notice the tenfold decrease achieved on passing from the "starting" U20-NACP material, to its Nano & TG counterparts, shown, in this manuscript, to possess a relatively high NUE, if compared to soluble ammonium phosphates.

Material	Cost per gram (€ g^{-1})	Cost per mol of N (€ mol N^{-1})
U20-NACP	1.033	177.9
TG-U13.5-NACP	0.085	17.3
Nano-U-ACP	0.080	17.3
Diammonium phosphate	0.038	2.5
Monoammonium phosphate	0.032	3.7

Supplementary Figures

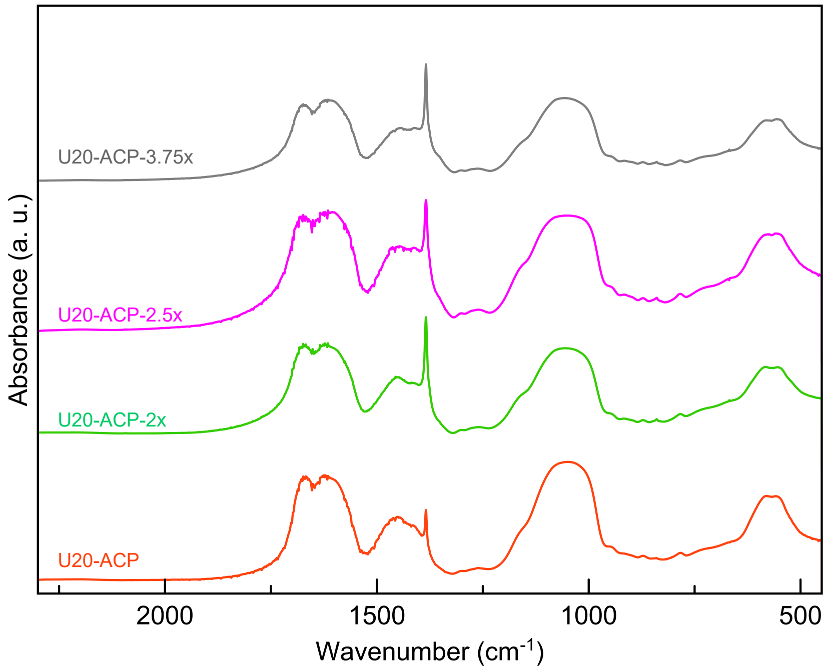

Figure S1. FTIR spectra of U20-NACP, U20-NACP-2x, U20-NACP-2.5x, U20-NACP-3.75x materials. All spectra show the typical bands related to ACP, as well as the characteristics bands of nitrate ions (1384 cm^{-1}) and urea (1668 cm^{-1} and 1465 cm^{-1}).

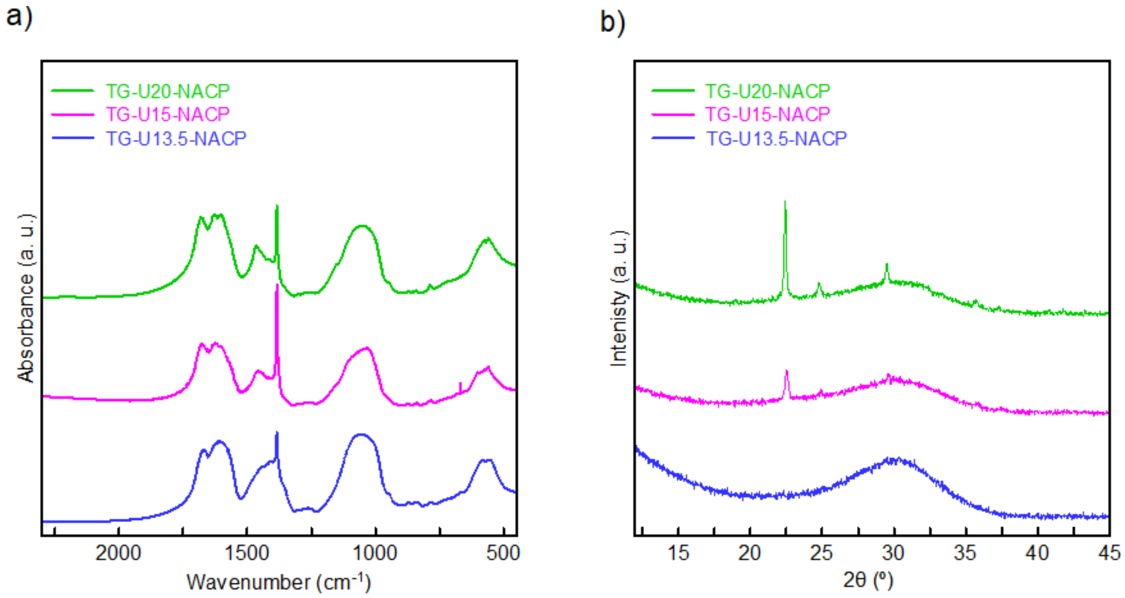

Figure S2. FTIR spectra (a) and XRD patterns (b) of the urea-doped materials prepared with technical grade reagents and nominal urea content of 20, 15 and 13.5 w%: TG-U20-NACP (green), TG-U15-NACP (magenta) and TG-U13.5-NACP (blue) materials. All spectra show the typical bands related to ACP, as well as the characteristics bands of nitrate ions (1384 cm^{-1}) and urea (1668 cm^{-1} and 1465 cm^{-1}). Only TG-U13.5-NACP is entirely amorphous, the other two samples showing co-precipitation of crystalline urea.

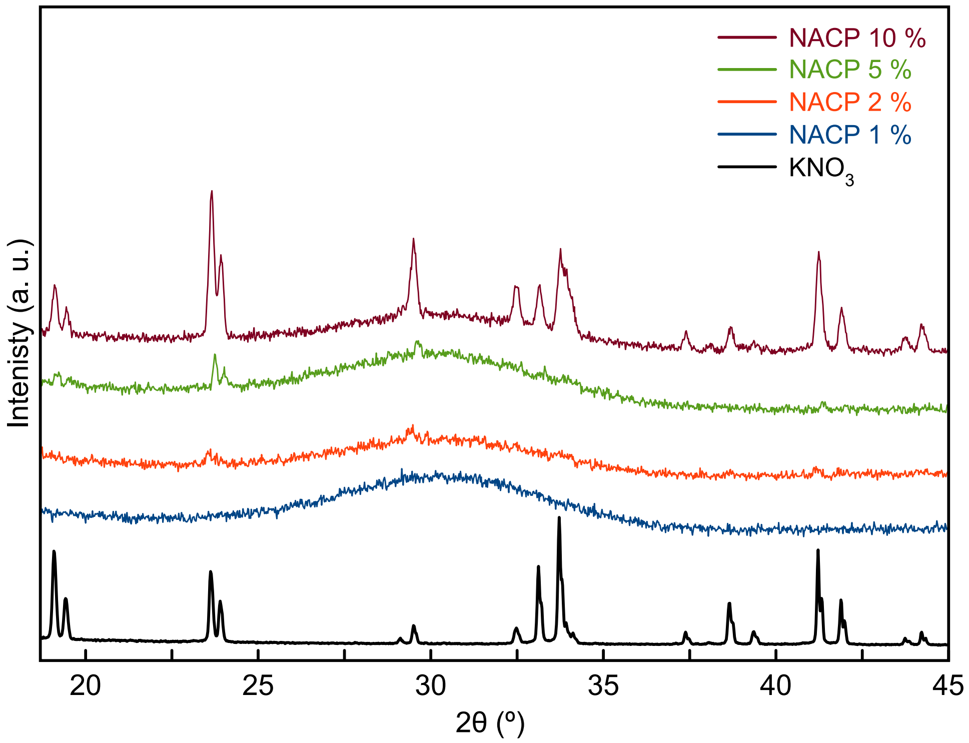

Figure S3. XRPD patterns of the ACP materials, post-synthetically doped with nitrate ions: nitrate content: 1 % (blue line), 2 % (orange) 5 % (green) and 10 % (brown). The XRPD pattern of pure KNO_3 is shown in black. Characteristics Bragg peaks of KNO_3 can be barely appreciated when a 2 % of nitrate is added, being more evident at the 5 and 10 % levels.

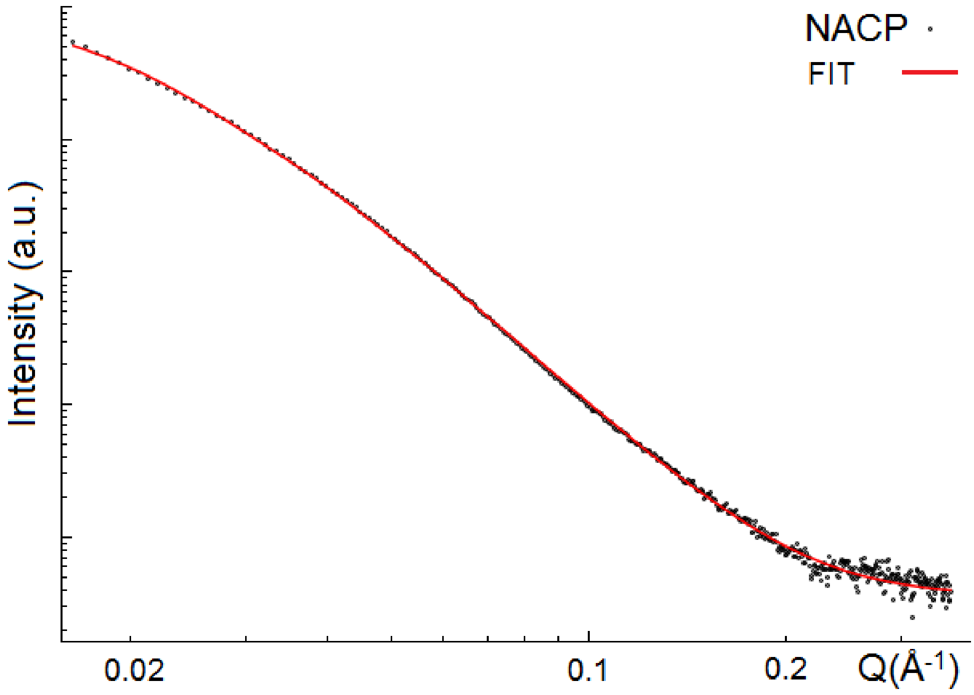

Figure S4. SAXS data and relative fit for the NACP-1x sample, measured prior to urea post-synthetic functionalization. The log-log plot shows the intensity vs. Q dependence, where $Q = 4\pi\sin\theta/\lambda$, 2θ being the scattering angle, λ the wavelength of the X-ray radiation used (here, λ = 1.5418 Å). Data were fitted by a population of spherical nanoparticles following a number-based lognormal distribution of diameters with average diameter of 6.8 nm and relative polydispersity of 63%.

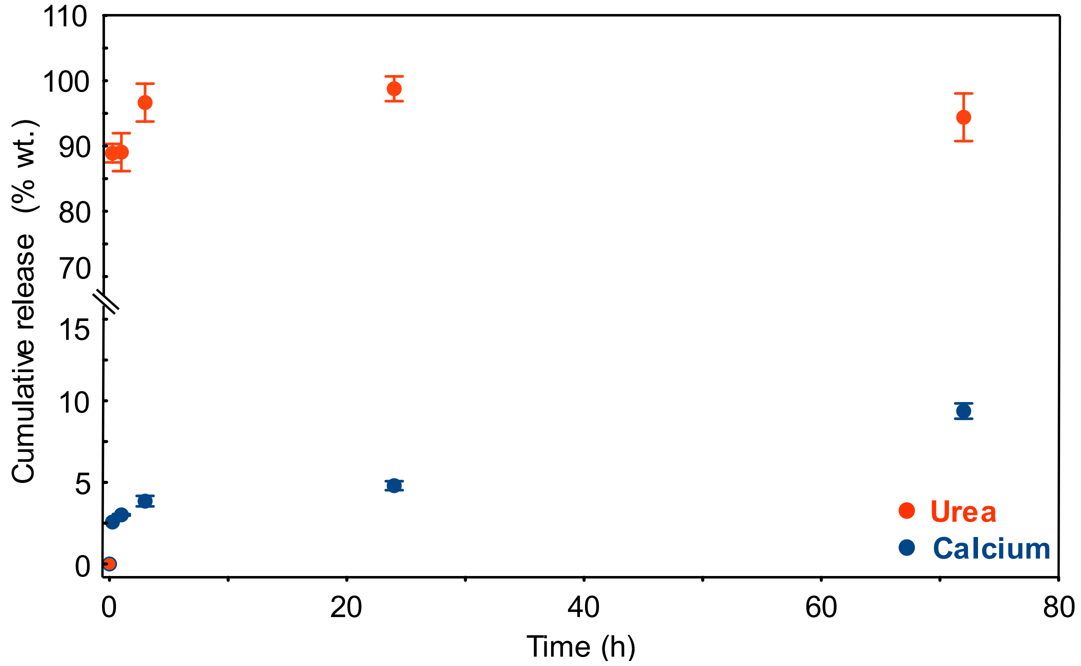

Figure S5. Urea and calcium release profiles from Nano-U-ACP under suspension in water at room temperature (Urea, red circles, Calcium ions, blue circles). Taking into account that, in the presence of some complexing citrate generated by nanoparticle dissolution, the concentration the “truly free” Ca^{2+} ions values may be somewhat underestimated, a slightly larger overall cumulative release of phosphate is favored.

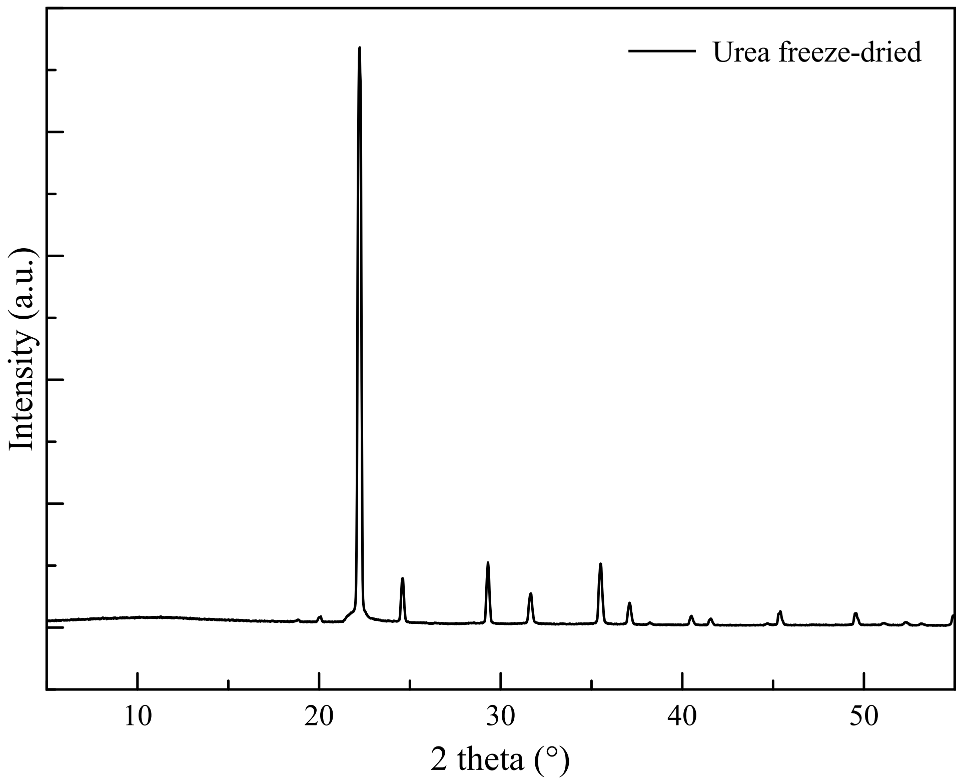

Figure S6: X-Ray powder diffractogram of lyophilized urea slurry. Urea is fully crystalline.

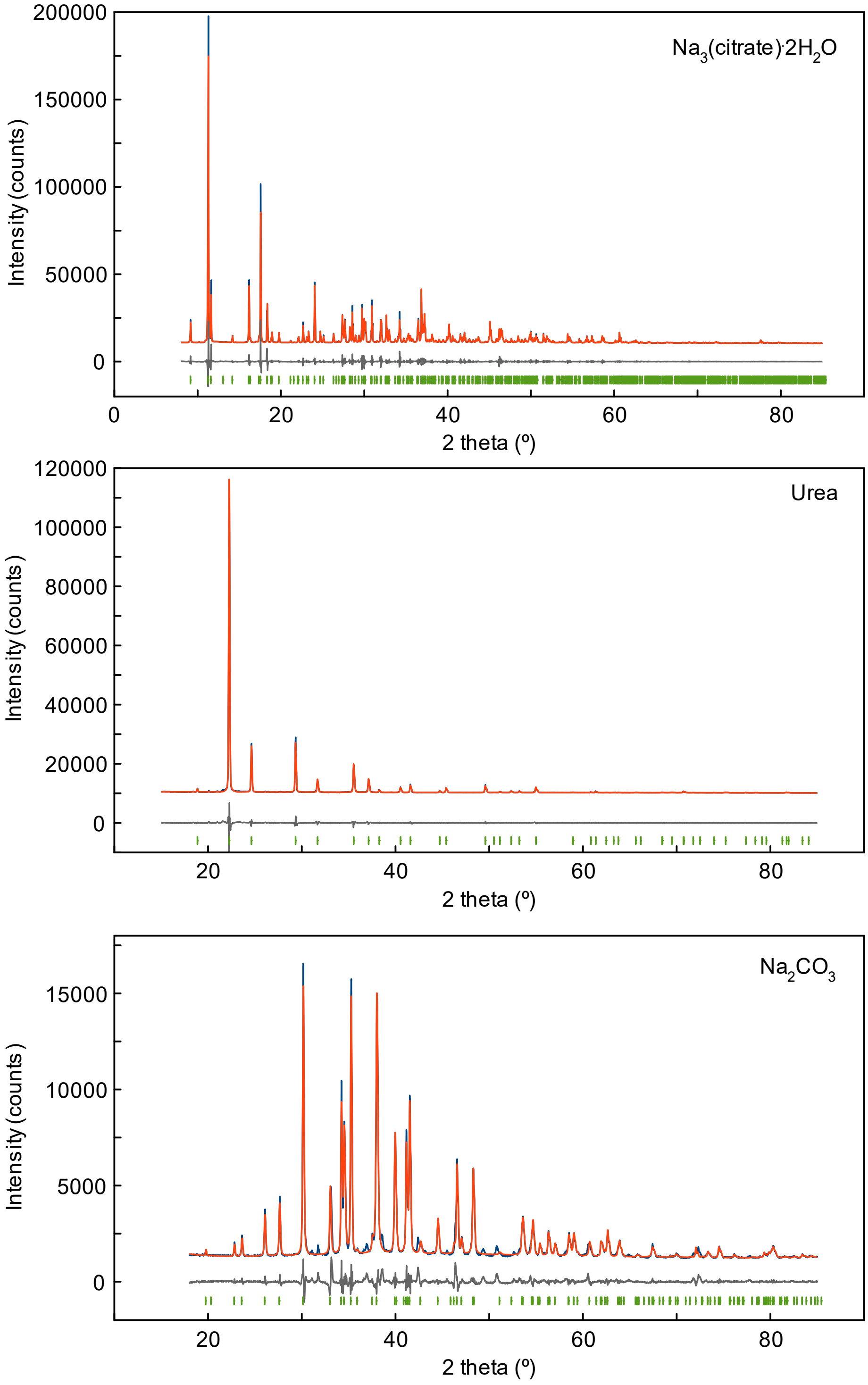

Figure S7. Graphical result of the identification of the crystalline phases on technical grade reagents by the Le Bail method performed on (top to bottom): Na_3(citrate)·$2H_2O$, urea and Na_2CO_3, in terms of experimental, calculated and difference traces (blue, red and grey, respectively). The green markers at the bottom indicate the position of the Bragg reflection for each crystalline phase.

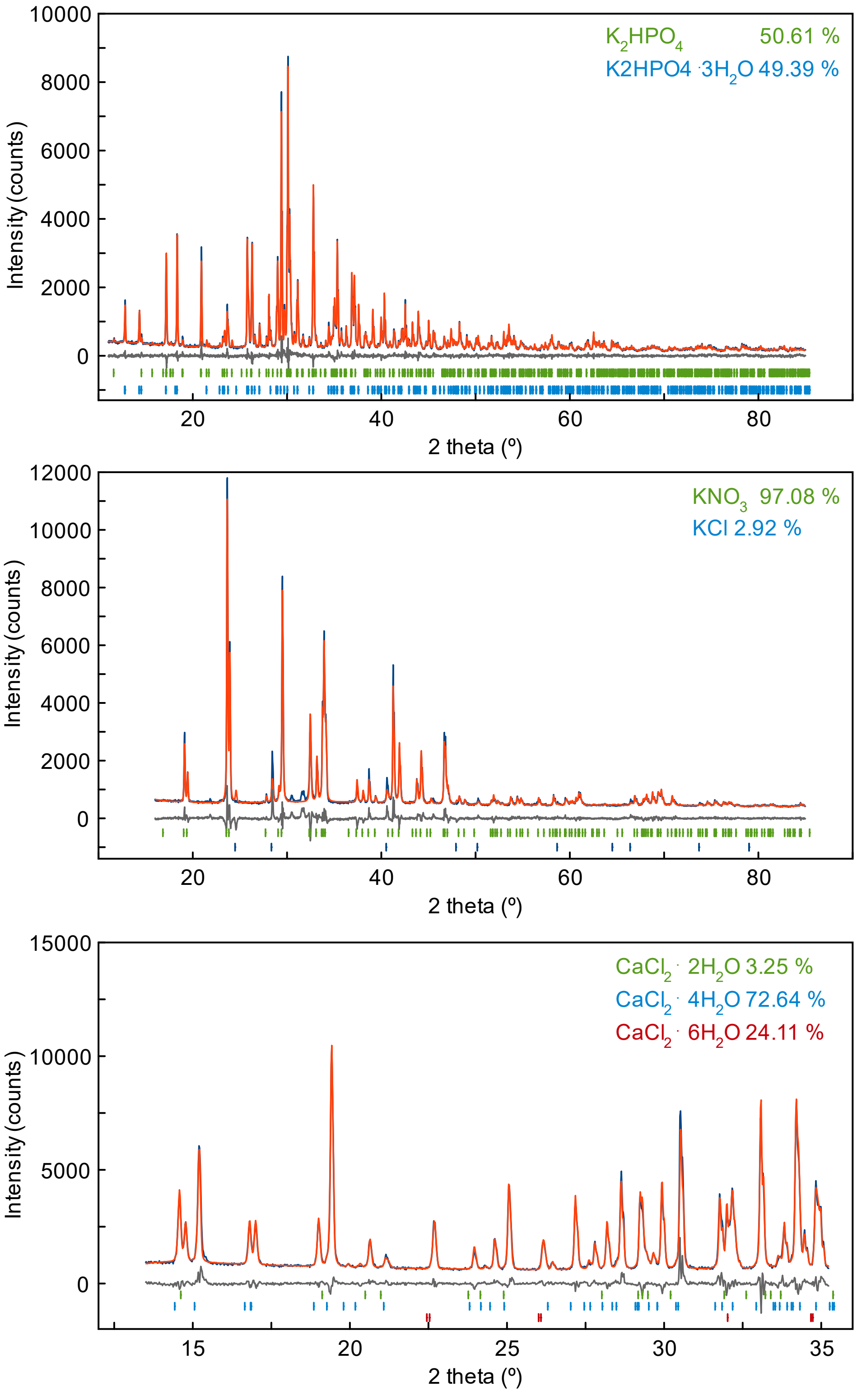

Figure S8. Graphical result of the quantification of the crystalline phases by Rietveld refinement carried out on (top to bottom): K_2HPO_4 (technical grade), KNO_3 (technical grade) and $CaCl_2$ (technical grade) in terms of observed, calculated and difference traces (blue, red and grey, respectively). The markers at the bottom indicate the position of the Bragg reflection for each crystalline phase.

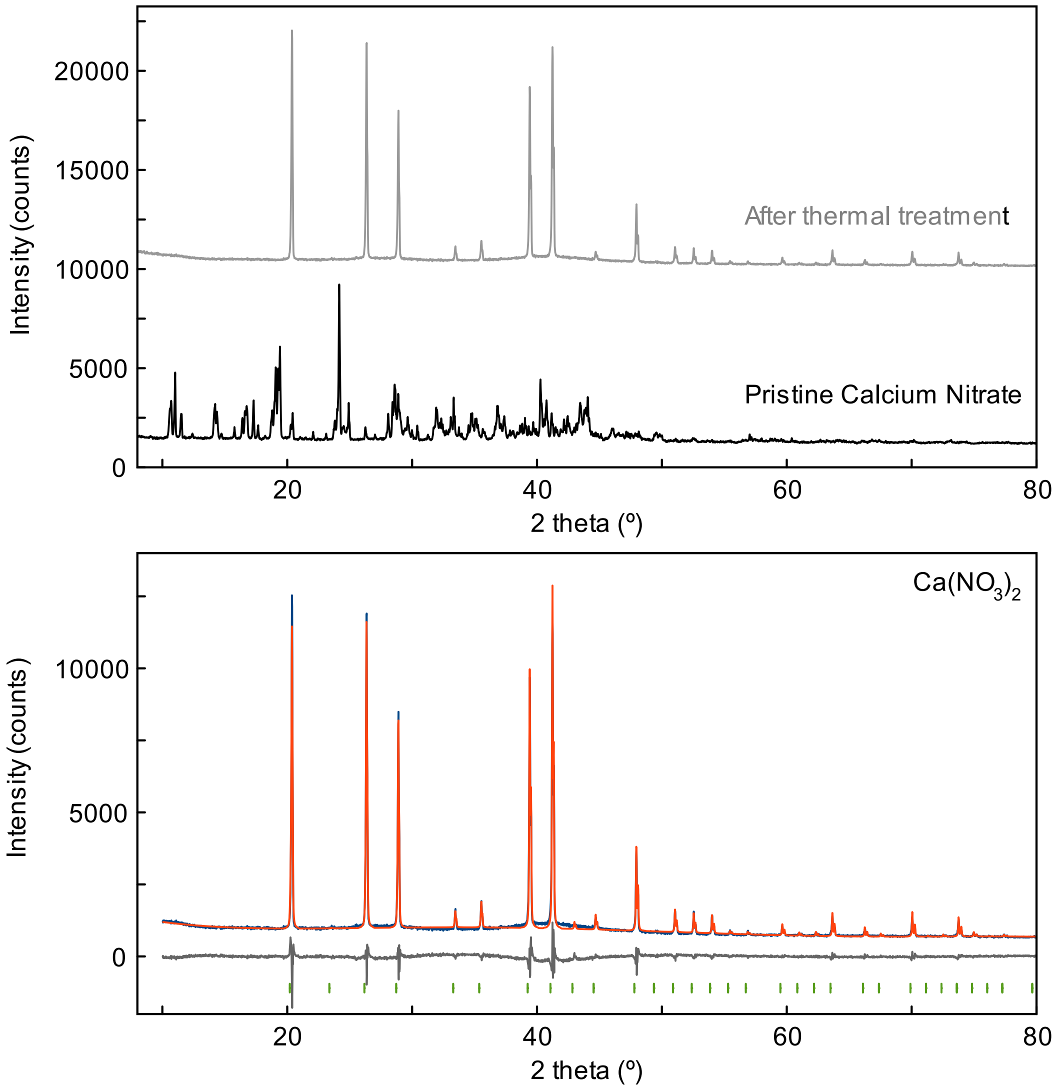

Figure S9. Top: X-Ray Powder pattern of pristine calcium nitrate reagent (technical grade) as received (black curve) and after thermal treatment at 300 ºC during 2 h (grey curve); bottom: graphical result of the identification of the crystalline phases by the Le Bail method of $Ca(NO_3)_2$ after thermal treatment, in terms of observed, calculated and difference traces (blue, red and grey, respectively). The markers in green at the bottom indicate the position of the Bragg reflection for the crystalline phase.

Supplementary Text

S1. Assessment of the phase composition of technical grade reagents by XRPD*:*

Before any synthesis using technical grade (TG) reagents was conducted, their purity was assessed (Step 3 of Scheme 2 in the main text) by XRPD on data measured in the 5-85° angular range (see Figures S7, S8 and S9). For calcium chloride, due to its elevated hygroscopicity, XRPD data were collected in a shorter range (13-36º). The purity of crystalline TG reagents was investigated by means of the Le Bail fitting method.[1] For sodium citrate dihydrate, $Na_3(C_6H_5O_7)\cdot 2H_2O$ (CSD code: UMOGAE), sodium carbonate, Na_2CO_3 (ICSD code: 95549) and urea, $CO(NH_2)_2$ (COD code: 1008776) the analysis confirmed the absence of unindexed peaks[2] The TG reagent "potassium phosphate dibasic" contained a mixture of the K_2HPO_4[3] and $K_2HPO_4\cdot 3H_2O$[4] crystalline phases. The TG reagent "potassium nitrate" was composed by a mixture of KNO_3 (ICSD code: 10289) and KCl (ICSD code: 165593). The TG reagent "calcium chloride" contained three different calcium chloride hydrates: $CaCl_2\cdot 2H_2O$ (COD code: 1001835), $CaCl_2\cdot 4H_2O$ (ISCD code: 8061) and $CaCl_2\cdot 6H_2O$ (COD code: 1001770). For these mixtures, the quantification of the crystal phase content was performed by the Rietveld method.[5] Results are provided in the insets of Figure S8. The TG reagent "calcium nitrate" contained a complex mixture of hydrates and its purity was gravimetrically determined. 2.510 g were heated in an oven from room temperature to 300 °C with a 10 º min^{-1} ramp and kept at 300 °C for 2 h, until anhydrous $Ca(NO_3)$ was obtained, whose phase purity was certified by XRPD (ICSD code: 52351). The water content was then estimated from the measured weight loss**.**

S2. Optimization of synthesis and post-synthetic N-doping of ACP nanoparticles prepared with technical grade reagents and tap water.

Once the best conditions for the preparation of Nitrogen-doped ACP nanomaterials have been determined, we studied the feasibility of carrying out the same procedure by using technical grade reagents. As these inexpensive technical grade reagents may have suffered uncontrolled storage conditions and can contain non-negligible amounts of materials with a different composition than the nominal one, we firstly assessed their phase purity, or their phase composition by XRPD (Figures S7-S9). The chemical formula and composition calculated for each starting material are shown in Table S3. The purity of urea, sodium citrate dihydrate and sodium carbonate compounds was easily confirmed (Figure S7). On the other hand, different hydrated forms or presence of impurities were identified in the other salts. The quantification

of the different phases (Figure S8), present in the potassium phosphate dibasic, potassium nitrate and calcium chloride raw materials, provided the chemical compositions illustrated in Table S3. Finally, as the XRPD pattern of calcium nitrate showed a complex mixture of different hydrates, its water content was determined gravimetrically, after applying a thermal treatment leading a pure anhydrous crystalline phase (Figure S9).

After the correct compositions of the different technical grade reagents were determined, these raw ingredients were used to prepare new doped-ACP materials by means of the post-synthetic doping approach (labelled as TG-U*a*-NACP powders, see Scheme 1). Upon adopting the optimized conditions from the analytical grade reagents tests (U20-NACP-2x), TG-U20-NACP exhibited a small co-precipitation of crystalline urea. Reducing the concentration of urea to 13.5 wt% enabled to obtain an entirely amorphous material, both nitrate ions (visible in the IR spectrum by the characteristic peak at 1384 cm^{-1}) and urea molecules (IR bands falling at 1668 cm^{-1} and 1465 cm^{-1}, Figure S2). The whole N content determined by elemental analysis for TG-U13.5-NACP was of 6.8 wt%, with a mere 1.3 wt% deviation from the reference U20-NACP-2x material.

Considering that all urea employed during the preparation (13.5%), is incorporated/adsorbed in/on the nanoparticles, the content of nitrate can be estimated around 2.2 wt%, a value not too distant from that reported by us for the one-pot doping approach (2.5 wt%).[a] Nevertheless, the efficiency of nitrate doping (calculated as the relation between the mass of nitrate in the material and total mass of nitrate added) is only of 3.42 wt %, being 96.58 % of added nitrate lost during the washing process.

Prompted by these promising results with urea and with the aim to increase the efficiency of nitrate ions doping, new attempts were also performed to incorporate NO_3^- within ACP by means of a post-synthetic functionalization process. However, XRPD of the nitrate-doped hybrid materials shows that the usage of a mass fraction of potassium nitrate higher than 2% leads to the undesired co-precipitation of crystalline KNO_3 (Figure S3). These findings highlight the higher affinity of urea than nitrate, toward ACP nanoparticle decoration.

S3. SAXS experiment

A small aliquot of the NACP-1x sample (used as the amorphous calcium phosphate material for the post-synthetic urea doping) was analyzed by Small Angle X-ray Scattering (SAXS) for particle size and shape determination. The SAXS measurement was performed at Aarhus University using an in-house SAXS instrument[6] equipped with a rotating Cu anode source, side-by-side Montel multilayer mirrors for beam monochromatization and focusing, and a

Vantec 500 (Bruker AXS) detector. The collimation system consists of two-pinholes, where the one close to the sample is a scatterless pinhole with edges of Ge crystal.[7,8] The powdered sample was dispersed on acetate Scotch tape and mounted in the beam in the integrated vacuum of the SAXS instrument. A piece of the same tape was measured and subtracted as background. The SUPERSAXS program package (in-house developed - C.L.P. Oliveira and J.S. Pedersen, unpublished) was used for the data reduction.

S4. DSE modelling of SAXS data

The NACP-1x SAXS signal was analyzed through a total scattering method based on the Debye scattering equation (DSE) using the DebUsSy Suite[9] (code availability. The *DebUsSy* program suite (freely available at *http://debussy.sourceforge.net*) performs calculations using atomistic models of nanoparticles (NPs) and a bottom-up approach to generate populations of NPs of different shapes and increasing sizes. DSE enables the computation of the total X-ray scattering pattern of randomly oriented and non-interacting particles from the set of interatomic distances calculated from the atomistic model.[9–11] Firstly, a population of NPs of spherical shape with diameters up to 20 nm was built. Pseudo-multiplicities *vs* equi-spaced pair distances were calculated and encoded in databases, according to a Gaussian sampling of interatomic distances to speed up calculations.[12] The calculated pattern is then optimized (monitoring the goodness of fit - GoF = square root of reduced χ^2) against the experimental data using a simplex minimization algorithm by adjusting the size parameters, namely the (number-based) average diameter and standard deviation of a univariate lognormal distribution function. This modelling approach, typically applied to Wide Angle X-ray Total Scattering data of nanocrystalline materials, can be effectively applied in the small angle region, provided that interparticle interactions can be reasonably neglected.[13–15] In the specific case of NACP-1x data, the DSE analysis was performed by assuming that *i)* in the Q range typical of SAXS experiments, the total scattering pattern is not affected by atomic-scale features; *ii)* the atomistic model of NPs with the crystal structure of hydroxyapatite (hexagonal crystal structure $P6_3/m$)[16] can reasonably account for the *average* electron density of ACP, as a suitable structural model for ACP does not exist.

The DSE best fit (GoF =3.158, Figure S4) suggests that primary particles of the NACP-1x sample exhibit spherical shape with average diameter of 6.8 nm and a size dispersion of 63% (lognormal number-based distribution). The corresponding mass-based values are 17.8 nm (average diameter) and 53% (size dispersion).

References for Supporting Information File

1. Le Bail, A. Whole powder pattern decomposition methods and applications: A retrospection. *Powder Diffr.* **20**, 316–326 (2005).
2. Le Bail, A., Duroy, H. & FourQuet, J. L. Ab-initio structure determination of LiSbWO6 by X-ray powder diffraction. *Mat. Res. Bull.* **23**, 447–452 (1988).
3. Lis, T. Isomorphous crystals: K2HPO4 and K5Na(HPO4)3. *Acta Crystallogr. Sect. C Cryst. Struct. Commun.* **50**, 484–487 (1994).
4. Baran, J., Lis, T. & Ratajczak, H. Structure and polarized IR spectra of the K2HPO4·3H2O crystal. *J. Mol. Struct.* **195**, 159–174 (1989).
5. Rietveld, H. M. A profile refinement method for nuclear and magnetic structures. *J. Appl. Crystallogr.* **2**, 65–71 (1969).
6. Pedersen, J. S. A flux- and background-optimized version of the NanoSTAR small-angle X-ray scattering camera for solution scattering. *J. Appl. Crystallogr.* **37**, 369–380 (2004).
7. Li, Y., Beck, R., Huang, T., Choi, M. C. & Divinagracia, M. Scatterless hybrid metal–single-crystal slit for small-angle X-ray scattering and high-resolution X-ray diffraction. *J. Appl. Crystallogr.* **41**, 1134–1139 (2008).
8. Pedersen, J. S. X-ray analyzing system for X-ray scattering analysis. (2018).
9. Cervellino, A., Frison, R., Bertolotti, F. & Guagliardi, A. DEBUSSY 2.0: The new release of a Debye user system for nanocrystalline and/or disordered materials. *J. Appl. Crystallogr.* **48**, 2026–2032 (2015).
10. Bertolotti, F., Moscheni, D., Guagliardi, A. & Masciocchi, N. When Crystals Go Nano – The Role of Advanced X-ray Total Scattering Methods in Nanotechnology. *Eur. J. Inorg. Chem.* **2018**, 3789–3803 (2018).
11. Debye, P. Zerstreuung von Röntgenstrahlen. *Ann. Phys.* **351**, 809–823 (1915).
12. Cervellino, A., Giannini, C. & Guagliardi, A. On the efficient evaluation of Fourier patterns for nanoparticles and clusters. *J. Comput. Chem.* **27**, 995–1008 (2006).
13. Gordon, T. R. *et al.* Characterization of Shape and Monodispersity of Anisotropic Nanocrystals through Atomistic X-ray Scattering Simulation. *Chem. Mater.* **27**, 2502–2506 (2015).
14. Moscheni, D. *et al.* Size-Dependent Fault-Driven Relaxation and Faceting in Zincblende CdSe Colloidal Quantum Dots. *ACS Nano* **12**, 12558–12570 (2018).
15. Bertolotti, F. *et al.* On the amorphous layer in bone mineral and biomimetic apatite: a

combined small- and wide-angle X-ray scattering analysis. *Acta Biomater.* **In press**, (2020).

16. Kay, M. I., Young, R. A. & Posner, A. S. Crystal structure of hydroxyapatite. *Nature* **204**, 1050–1052 (1964).
